# Chronic HBV infection impairs the glucose metabolism and effector function of NK cells via HBsAg/IL-15/mTOR axis

**DOI:** 10.1038/s41419-025-08069-y

**Published:** 2025-10-13

**Authors:** Yating Yu, Zixuan Wang, Ailu Yang, Yucan Wang, Cuiping Bao, Li Zhuo, Qiuju Han, Huajun Zhao, Jian Zhang

**Affiliations:** 1 State Key Laboratory of Discovery and Utilization of Functional Components in Traditional Chinese Medicine, Institute of Immunopharmaceutical Sciences, School of Pharmaceutical Sciences, Shandong University, Jinan, Shandong China; 2 State Key Laboratory of Microbial Technology, Institute of Microbial Technology, Shandong University, Qingdao, Shandong China

**Keywords:** Viral hepatitis, Viral hepatitis

## Abstract

Natural killer (NK) cell function is impaired in patients with chronic hepatitis B (CHB) infection; however, the underlying mechanisms are not fully understood. Here, we collected the blood samples from healthy donors (HDs) and patients with CHB, and then analyzed the characteristics of NK cells by RNA-seq analysis, flow cytometry, Seahorse assay. HBV-carrier mice were used to confirm the findings in vivo. We found that the dysfunction of NK cells in peripheral blood of patients with CHB was associated with the disturbance of glycolysis. Further investigation showed chronic HBV infection impaired the activation of mammalian target of rapamycin (mTOR) in NK cells, resulting in decreased expression of molecules involved in glycolysis, including HIF-1α and GLUT1. Mechanistically, we found that HBsAg suppressed IL-15-triggered mTOR activity by competitively binding to the IL-15 receptor β (IL-15Rβ, CD122) on NK cells, leading to the decreased expression of HIF-1α and its downstream genes. Significantly, HBsAg neutralizing antibody intravenous injection or mTOR agonist MHY1485 intraperitoneal injection restored the IL-15/mTOR signaling in NK cells of HBV-carrier mice, resulting in NK cell activation and HBV clearance. Further, transferring MHY1485-pretreated NK cells isolated from HBV-carrier mice displayed augmented anti-HBV effects in recipient HBV-carrier mice. These findings reveal a new mechanism by which chronic HBV infection induces NK cell dysfunction, and highlight the potential of mTOR activation and HBsAg clearance as therapeutic strategies for CHB treatment via recovering NK cell immune functions.

## Introduction

Chronic hepatitis B virus (HBV) infection is a global health problem. A total of 300 million people worldwide are suffered with chronic hepatitis B (CHB), which might gradually develop into liver fibrosis, cirrhosis, and even cancer [1, 2]. Chronic HBV infection is a dynamic process of the interactions between HBV replication and the host’s immune systems. As the first line of defense against virus infection, innate immune response is essential for the early control of HBV replication and the induction of adaptive immune response [3]. However, HBV has evolved multiple strategies for escaping immune surveillance, resulting in persistent HBV infection.

As an important component of innate immunity, NK cells can clear virally infected cells by releasing cytokines (IFN-γ, TNF-α), cytolytic granules (perforin, granzyme), and upregulating death-related receptors (AS-FASL, TRAIL-TRAILR) without major histocompatibility complex restriction [4]. However, chronic HBV infection significantly reduced the proportion of NK cells in the peripheral blood and induced NK cells into an exhaustion-like status, characterized by the increased inhibitory receptors (NKG2A, Siglec9, and Tim-3), decreased activating receptors (NKp30, NKG2D, and 2B4) and cytokines, and impaired cytotoxic activity [5–7], contributing to persistent HBV infection.

Immune responses need rapid and extensive changes of cellular metabolism in immune cells. Accelerated cellular metabolism is required to support the activities of immune cells, and the metabolic programs can be dominated to meet diverse energy demands [8]. It has been established that NK cells are primarily fueled by glucose, and glucose-driven glycolysis and oxidative phosphorylation (OXPHOS) are essential for NK cell effector functions [9, 10]. The basic metabolic rate of resting NK cells is low, maintaining only low levels of glycolysis and OXPHOS rates [11]. However, the stimulation of IL-2 alone or in combination with IL-15 significantly increases the rates of glycolysis and OXPHOS, which is necessary for NK cells cytotoxicity [12]. In contrast, the inhibition of glycolysis or mitochondrial activity significantly impairs NK cell-mediated anti-tumor and anti-rival responses [13, 14]. Previous study showed NK cells generated well-characterized metabolic changes upon virus infection [15–18]. Under the persistent HIV infection, NK cells showed mitochondrial dysfunction/structural alterations, characterized by defective OXPHOS and fragmented mitochondria, resulting in the decrease of IFN-γ production by NK cells and chronic HIV infection [15]. In addition, the acute MCMV infection reprogrammed the metabolic machinery of NK cells, manifested as the increased glycolysis, mitochondrial metabolism and nutrient uptake [16–18]. Nevertheless, whether NK cells dysfunction in chronic HBV infection is associated with glycolysis and OXPHOS metabolism is unknown.

The mechanistic target of rapamycin (mTOR) and the interactions between the adenosine monophosphate-activated protein kinase and hypoxia inducible factor-1 alpha (HIF-1α) regulate varieties of cellular processes, including gene transcription, protein translation and ribosome synthesis, therefore play crucial roles in cell growth, autophagy and metabolism in response to various extracellular signals such as variations in nutrients, energy and growth factors [19–21]. mTOR is also involved in the metabolisms of glucose, lipid, and amino acid in immune cells, serving as an important metabolic checkpoint [22–24]. mTOR is activated in NK cells exposed to IL-15, which boosts bioenergetic metabolism and contributes to NK cell-mediated anti-tumor activity [25–27]. However, it is still unclear whether the dysfunction of NK cells in chronic HBV infection is related to mTOR signaling.

In this study, we found chronic HBV infection inhibited IL-15-triggered mTOR signal transduction in NK cells, leading to decreased glycolysis and impaired NK cell function. Furthermore, HBsAg was confirmed to competitively bind to the IL-15Rβ on the surface of NK cells, while mTOR agonist can improve NK cell function and promote anti-HBV clearance.

## Materials And Methods

### Animals and reagents

Male C57BL/6J mice (5–6-weeks old) were obtained from Beijing HFK Bioscience Co., Ltd. (Beijing, China). Recombinant hepatitis B surface antigen (HBsAg; H. polymorpha) were purchased from Dalian Hissen Bio-pharm. Co., Ltd. (Dalian, China). Recombinant hepatitis B e antigen (HBeAg) was purchased from Beijing Key-bio Biotech. Co., Ltd. (Beijing, China). Recombinant adeno-associated virus (rAAV)8-HBV1.3 vectors containing 1.3-fold HBV genomes (genotype D, subtype ayw) were purchased from PackGene Biotech (Guangzhou, China).

### Patients and healthy donors

Peripheral blood samples from patients with CHB and healthy donors (HDs) were collected from the Second Hospital of Shandong University. The inclusion criteria and clinical characteristics of the patients with CHB and HDs were listed in Supplementary Table 1.

### Isolation of human PBMCs and NK cells

Human PBMCs were separated from peripheral blood by density gradient centrifugation at room temperature (RT) using Human Lymphocyte Separation Medium (Solarbio, Beijing, China). NK cells from PBMCs were sorted according to the instructions of MojoSort™ Human NK Cell Isolation Kit (Biolegend, USA). Cells were then resuspended in RPMI-1640 medium (Biological Industries, Israel) containing 10% Fetal Bovine Serum (FBS, Biological Industries, Israel) and supplemented with 100 U/mL recombinant human IL-2 (rhIL-2, Changchun Institute of Biological Products, Changchun, China).

### Cell line culture

NK-92 cell line was purchased from ATCC and cultured in α-minimum essential medium (Biological Industries, Israel) with 10% FBS, 12.5% horse serum, 100 U/mL rhIL-2, 0.055 mM β-mercaptoethanol, and 0.02 mM folic acid. HepG2.2.15 and HEK293T cells were maintained in our laboratory and cultured in Dulbecco’s modification of Eagle’s medium (Biological Industries, Israel) supplemented with 10% FBS. All cells were maintained at 37 °C in a 5% CO_2_ incubator.

### Flow cytometry analysis

Fc receptors were blockaded by incubating with 1× phosphate-buffered saline containing 10% rat or mouse serum for 30 min. For cell surface markers, cells were stained with the indicated fluorescently labeled antibodies for 30 min. For the analysis of intracellular molecules, cells were fixed using Foxp3 Transcription Factor Fixation/Permeation Concentrate (eBioscience, USA), and then stained with intracellular Phospho-mTOR mAb and Phospho-P70S6K mAb [28] or other antibodies. For analysis of intracellular cytokines, cells were stimulated with 30 ng/mL Phorbol 12-Myristate 13-Acetate (PMA, Beyotime, Shanghai, China) and 1 μg/mL ionomycin (Beyotime, Shanghai, China) in the presence of 100 U/mL rhIL-2 and 5 μg/mL brefeldin A (BioLegend, USA) for 4 h at 37 °C, and then were done in the same way as the intracellular molecular. Data were collected using a FACSymphony A3 or FACSCelesta system (BD Biosciences, USA) and analyzed with FlowJo software (FlowJo, USA). The antibodies were shown in Supplementary Table 2.

### RNA, DNA isolation, and quantitative RT-qPCR

Total RNA was extracted with TRIzol Reagent (CWBIO, Beijing, China) according to the manufacturer’s instructions, and cDNA was synthesized using HiFiscript cDNA synthesis kit (CWBIO, Beijing, China). DNA was extracted using TIANamp Genomic DNA kit (TIANGEN, Beijing, China). 2 × UltraSYBR Mixture (CWBIO, Beijing, China) was used for RT-qPCR according to the manufacturer’s instructions. The primer sequences are shown in Supplementary Table 3.

### Western blotting

Cells were lysed with RIPA lysis buffer (Beyotime, Shanghai, China) supplemented with protease inhibitor cocktail (Bimake, USA) and phosphatase inhibitor cocktail (Bimake, USA). Denatured proteins were separated by SDS-PAGE and transferred to polyvinylidene difluoride membranes (Millipore, USA). The membranes were blocked with 5% skim milk in TBS buffer at RT for 1 h and then incubated overnight with Phospho-mTOR mAb and Phospho-P70S6K mAb [29] or other antibodies at 4 °C. The antibodies were shown in Supplementary Table 4.

### HBV-carrier mouse model

HBV-carrier mouse model was generated through intravenous injection of 1 × 10^10^ vector genome equivalent of rAAV8-HBV1.3 as previously described [30]. At 6 weeks post-injection, serum HBsAg was detected, and mice with HBsAg level > 500 IU/mL were defined as HBV-carrier mice. Then, these mice were randomly divided into different groups for administration.

### Statistical analysis

Statistical analysis was performed using GraphPad Prism software. Differences between two groups were analyzed using the unpaired Student’s *t*-test for variables, and differences among multiple groups were analyzed using the two-way ANOVA for variables. The Pearson coefficient was used for analyzing correlation between variables. Data are presented as mean ± SEM. *p* < 0.05 was considered significant. *p < 0.05, **p < 0.01, ***p < 0.001, ****p < 0.0001.

## Results

### Chronic HBV infection impairs the glycolysis of NK cells

We collected the blood samples from HDs and patients with CHB that divided into HBeAg-positive and HBeAg-negative groups based on the new nomenclature [2]. Firstly, we analyzed the characterizations of CD3^−^ CD56^+^ NK cells in PBMCs using flow cytometry (Fig. S1A). Consistent with previous reports [28], the results showed that the proportion of NK cells was significantly reduced in patients with CHB compared to HDs (Fig. S1B), accompanied with the reduction of IFN-γ, granzyme B, and perforin (Fig. S1C–E), but no significant difference between HBeAg-positive and HBeAg-negative patients was shown. These results confirmed that chronic HBV infection reduces NK cell proportion and impairs NK cell function.

**Fig. 1:**
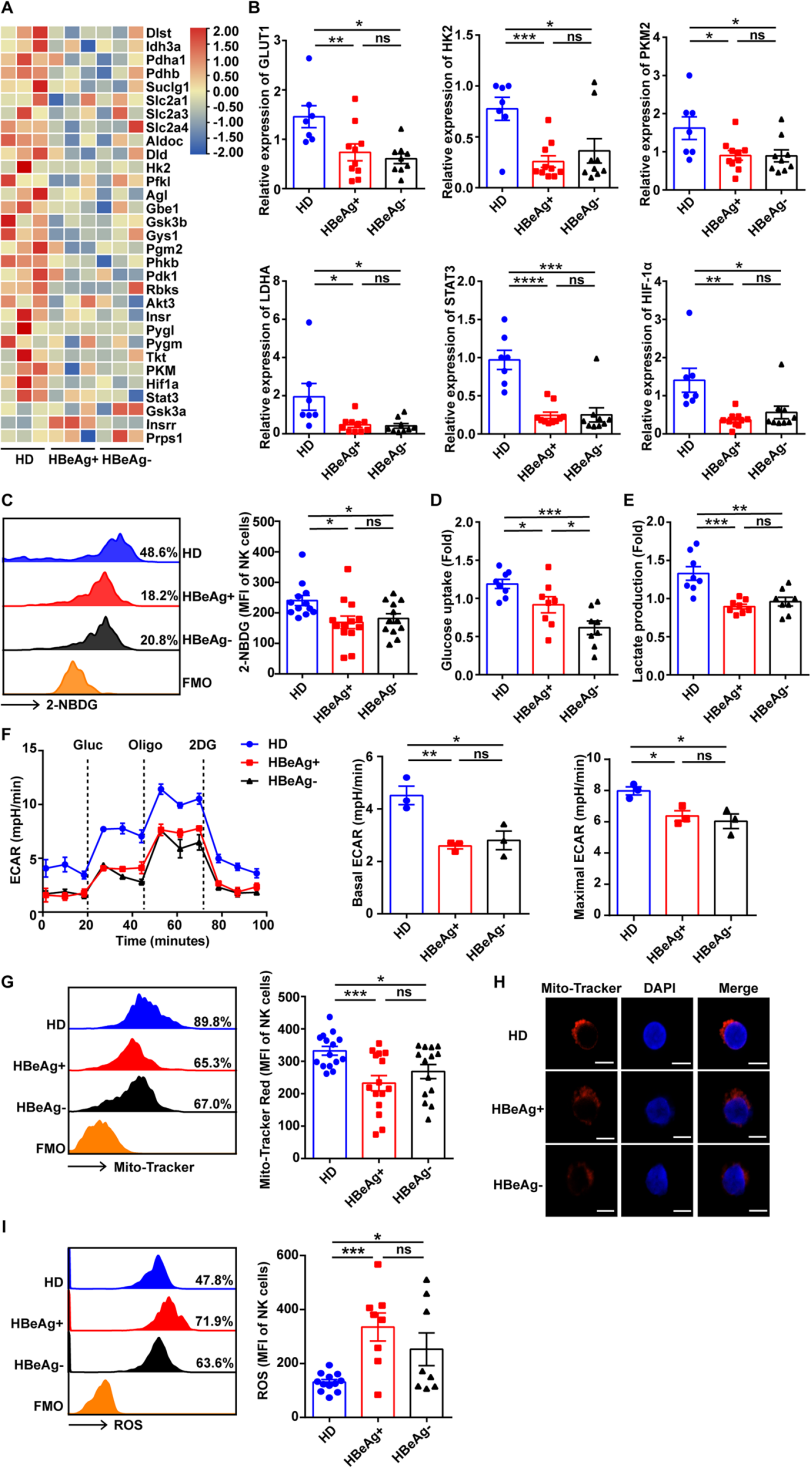
NK cells from patients with CHB exhibit reduced glycolysis and damaged mitochondrial activity. **A** NK cells were isolated from HDs, HBeAg^+^, and HBeAg^−^ patients with CHB for RNA-seq, heatmap showed differentially expressed genes associated with glycolysis. **B** Relative expression of mRNA levels of the indicated genes in NK cells isolated from HDs (*n* = 7), HBeAg^+^ (*n* = 10), and HBeAg^−^ (*n* = 9) patients with CHB was measured by RT-qPCR. **C** Representative histogram and mean fluorescence intensity (MFI) of 2-NBDG-stained NK cells from HDs (*n* = 12), HBeAg^+^ (*n* = 13), and HBeAg^−^ (*n* = 12) patients with CHB analyzed by flow cytometry. **D**, **E** NK cells isolated from HDs, HBeAg^+^, and HBeAg^−^ patients with CHB were cultured in vitro for 24 h, then the supernatant was collected, and relative glucose uptake (**D**) and lactate production (**E**) fold were detected. **F** NK cells isolated from HDs, HBeAg^+^, and HBeAg^−^ patients with CHB were stimulated with 500 U/mL IL-2 for 18 h, ECAR, basal ECAR, and maximal ECAR were measured upon addition of glucose (Gluc), oligomycin (Oligo), and 2-deoxy-D-glucose (2DG). **G** MFI of Mito-Tracker Red-stained cells in HDs, HBeAg^+^, and HBeAg^−^ patients with CHB was detected by flow cytometry. **H** Representative confocal microscopy images of NK cells isolated from HDs, HBeAg^+^, and HBeAg^−^ patients with CHB stained with Mito-Tracker (red) and DAPI (blue). The scale bars are all 5 μm. **I** MFI of ROS in NK cells from HDs, HBeAg^+^, and HBeAg^−^ patients with CHB was analyzed by flow cytometry. Differences between these groups were analyzed using the two-way ANOVA for variables. Data were presented as mean ± SEM. ns no significant. *p < 0.05, **p < 0.01, ***p < 0.001, ****p < 0.0001. SEM standard error of the mean.

To ensure the effector function, immune cells must take up large amounts of nutrients for cellular metabolism to maintain the huge energy demand. To identify the metabolic mechanisms involved in chronic HBV infection-elicited NK cell dysfunction, NK cells isolated from PBMCs of HDs and CHB patients were performed RNA-seq. We found many key genes associated with glucose metabolism such as GLUT1, HK2, PKM2, STAT3, and HIF1-α were downregulated in NK cells from patients with CHB compare to that from HDs (Fig. 1A), which was further confirmed by RT-qPCR (Fig. 1B). Meanwhile, the uptake ability 2-NBDG in NK cells from patients with CHB was significantly dampened compared to NK cells from HDs (Fig. 1C). And, the levels of glucose uptake and lactate production were decreased in NK cells from patients with CHB (Fig. 1D, E), accompanied with reduced ECAR (Fig. 1F). Furthermore, chronic HBV infection disturbed the mitochondrial membrane potential (Fig. 1G) and mitochondrial activity of NK cells (Fig. 1H), and increased the production of reactive oxygen species (ROS) (Fig. 1I). These results suggest that chronic HBV infection might damage NK cell function by interfering with glycolysis and disturbing mitochondrial function.

### Chronic HBV infection inhibits NK cell glycolysis via mTOR pathway

**Fig. 2:**
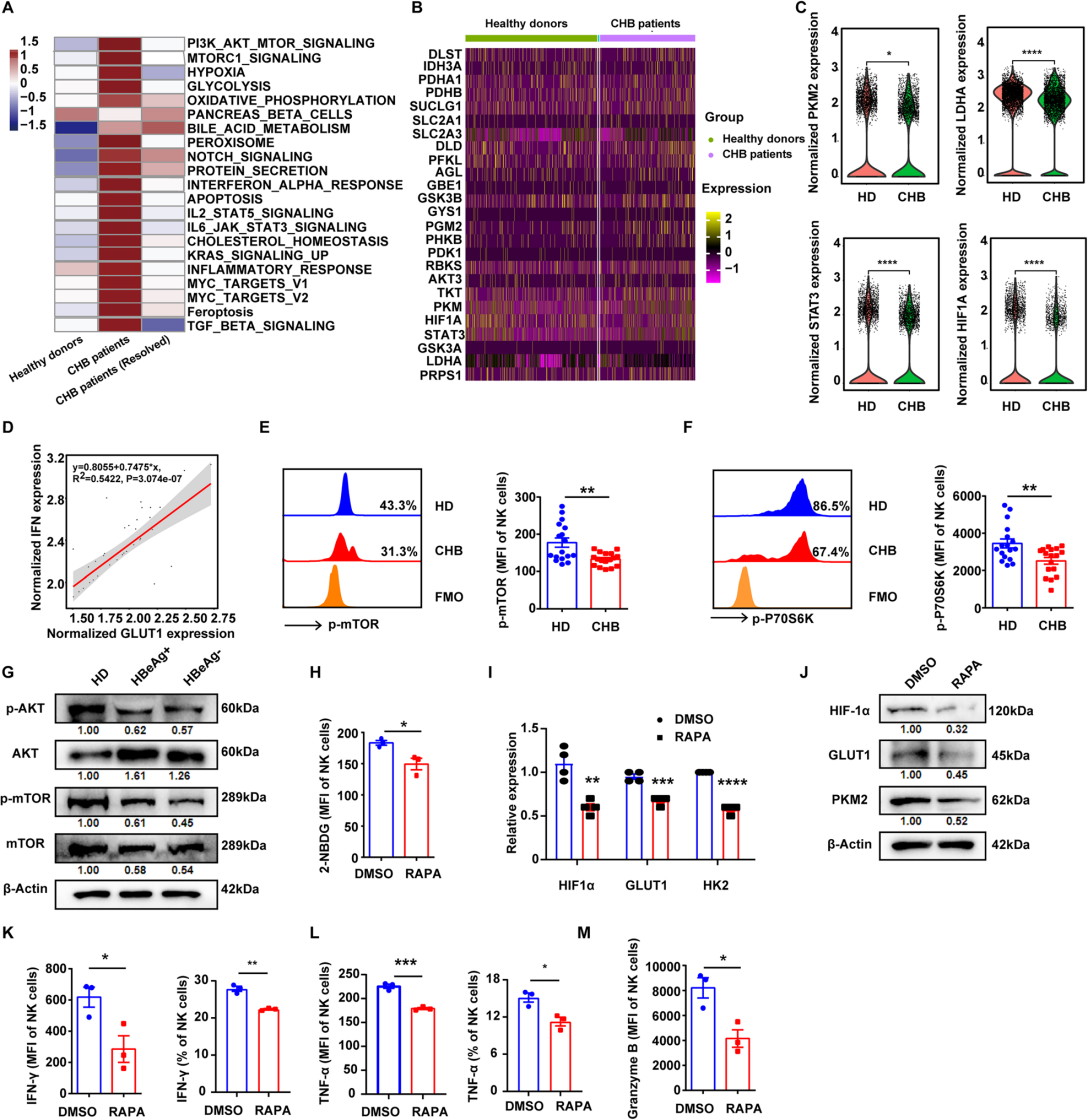
Chronic HBV infection inhibits mTOR signal pathway in NK cells. **A** Genes enrichment pathways of NK cells by intrahepatic single cell transcriptomics from public scRNA-seq database (GSE182159). **B** Landscape heatmap showing differentially expressed genes associated with glycolysis in hepatic NK cells between HDs and patients with CHB via scRNA-seq database (GSE182159). **C** The expression of PKM2, LDHA, STAT3 and HIF1A in hepatic NK cells from HDs and patients with CHB via scRNA-seq database (GSE182159). **D** The correlation between the levels IFN-γ and Glut1 in NK cells via scRNA-seq database (GSE182159). The solid line represented the linear regression fit, with the shaded region indicating the 95% confidence interval of the regression (R² = 0.5422). **E**, **F** The levels of phospho-mTOR (*p*-mTOR, **E**) and phospho-P70S6K (*p*-P70S6K, **F**) of NK cells in HDs and patients with CHB were measured by flow cytometry. **G** NK cells were isolated from HDs, HBeAg^+^, and HBeAg^−^ patients with CHB, and the indicated molecules were analyzed by western blotting. Band intensities were quantified using ImageJ software and normalized to HD group. **H** PBMCs from HDs were treated with 1 μM rapamycin for 6 h, then the level of 2-NBDG in NK cells was measured by flow cytometry. **I** NK cells isolated from HDs were treated with rapamycin for 6 h, then the expression of indicated molecules was measured by RT-qPCR. **J** NK cells isolated from HDs were treated with rapamycin for 6 h, then the expression of indicated molecules was measured by western blotting. Band intensities were quantified using ImageJ software and normalized to DMSO group. **K**–**M** PBMCs from HDs were treated with 1 μM rapamycin for 24 h, then the levels of IFN-γ (**K**), TNF-α (**L**), and granzyme B (**M**) of NK cells were measured by flow cytometry. Differences between two groups were analyzed using the unpaired Student’s *t*-test for variables. Data were presented as mean ± SEM of at least three independent experiments. *p < 0.05, **p < 0.01, ***p < 0.001, ****p < 0.0001. SEM standard error of the mean.

By analysis of a public scRNA-seq database (GSE182159) of intrahepatic single-cell transcriptomics in patients with CHB, we found that the differential genes between NK cells from patients with CHB and HDs or clinically cured patients with CHB were concentrated in glycolysis, OXPHOS, PI3K/AKT/mTOR, etc (Fig. 2A). Consistent with the phenomena we observed in NK cells from PBMCs, many key genes associated with glucose metabolism, including PKM2, LDHA, STAT3 and HIF1-α, were downregulated in hepatic NK cells from patients with CHB compare to that from HDs (Fig. 2B, C). We also detected a positive correlation between the IFN-γ secretion and Glut1 expression in NK cells (Fig. 2D, R^2^ = 0.5422), further indicating that chronic HBV infection-elicited NK cell dysfunction was due to the impaired glycolysis of NK cells. Emerging evidences suggest that mTOR is a key regulator of cellular metabolism and immune responses. Flow cytometry and western blotting analyses showed that the expression of *p*-AKT, *p*-mTOR, and *p*-P70S6K in NK cells of patients with CHB was significantly lower than that of HDs (Fig. 2E–G). In addition, mTOR inhibitor rapamycin observably inhibited the uptake capacity of 2-NBDG in NK cells (Fig. 2H) and the expression of molecules involved in glycolysis, such as HIF-1α and GLUT1 at both mRNA and protein levels in NK cells from HDs (Fig. 2I, J). Meanwhile, the production of IFN-γ, TNF-α, and granzyme B in NK cells was significantly downregulated by rapamycin treatment (Fig. 2K–M). These data indicate that chronic HBV infection interferes with NK cell glycolysis via inhibiting mTOR signaling pathway.

### HBsAg suppresses the glycolysis of NK cells by inhibiting IL-15-triggered mTOR activity

**Fig. 3:**
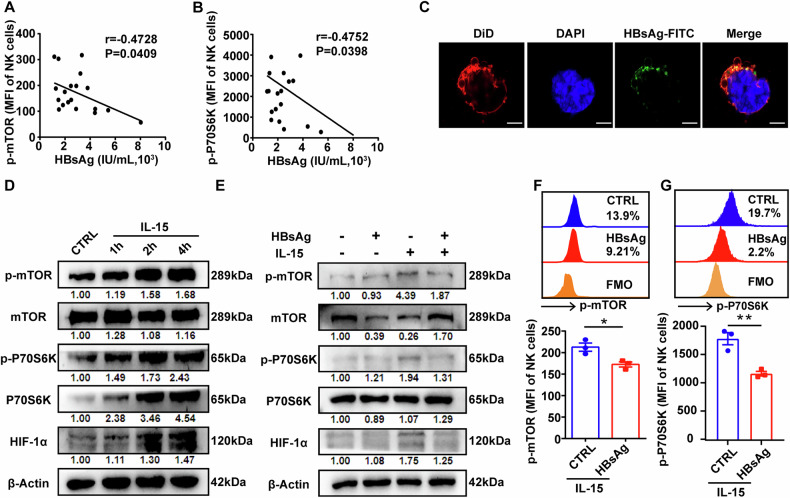
HBsAg inhibits IL-15-triggered mTOR signaling transduction in NK cells. **A**, **B** Correlation between the serum HBsAg level and MFI of *p*-mTOR (**A**) and *p*-P70S6K (**B**) in NK cells of HBeAg^−^ patients with CHB. **C** NK-92 cells incubated with 20 μg/mL HBsAg-FITC (green) for 24 h were stained with DiD (red) and DAPI (blue). Representative confocal microscopy images, and the scale bars are all 5 μm. **D** NK-92 cells were stimulated with 20 ng/mL IL-15 for 1, 2, 4 h, and then the indicated molecules were analyzed by western blotting. Band intensities were quantified using ImageJ software and normalized to CTRL group. **E** NK cells isolated from HDs were incubated with HBsAg for 24 h, and then stimulated with 20 ng/mL IL-15 for 1 h. The indicated molecules were analyzed by Western blotting. Band intensities were quantified using ImageJ software and normalized to CTRL group. **F**, **G** PBMCs from HDs were incubated with 20 μg/mL HBsAg for 24 h, then stimulated with 20 ng/mL IL-15 for 1 h. The levels of *p*-mTOR (**F**) and *p*-P70S6K (**G**) in NK cells were measured by flow cytometry. Differences between these two groups were analyzed using the unpaired Student’s *t*-test for variables. Data were presented as mean ± SEM of at least three independent experiments. *p < 0.05, **p < 0.01. SEM, standard error of the mean.

HBsAg is present in the serum of patients with CHB throughout the natural history of the chronic HBV infection, contributing to immune evasion and persistent immune tolerance of HBV [31]. We found a significant negative correlation between the serum levels of HBsAg and the levels of *p*-mTOR and *p*-P70S6K in NK cells from patients with CHB (Fig. 3A, B). Our previous studies showed that HBsAg could bind to the surface of NK cells [32]. Here, we confirmed again by confocal microscopy that FITC-labeled HBsAg could bind to the membrane of NK cells (Fig. 3C). However, HBsAg did not inhibit the mTOR signaling in NK-92 cells directly (Fig. S2A–C), nor significantly influence the uptake of 2-NBDG and mitochondrial membrane potential of NK-92 cells (Fig. S2D, E), indicating HBsAg would not directly affect the glycolysis of NK cells.

**Fig. 4:**
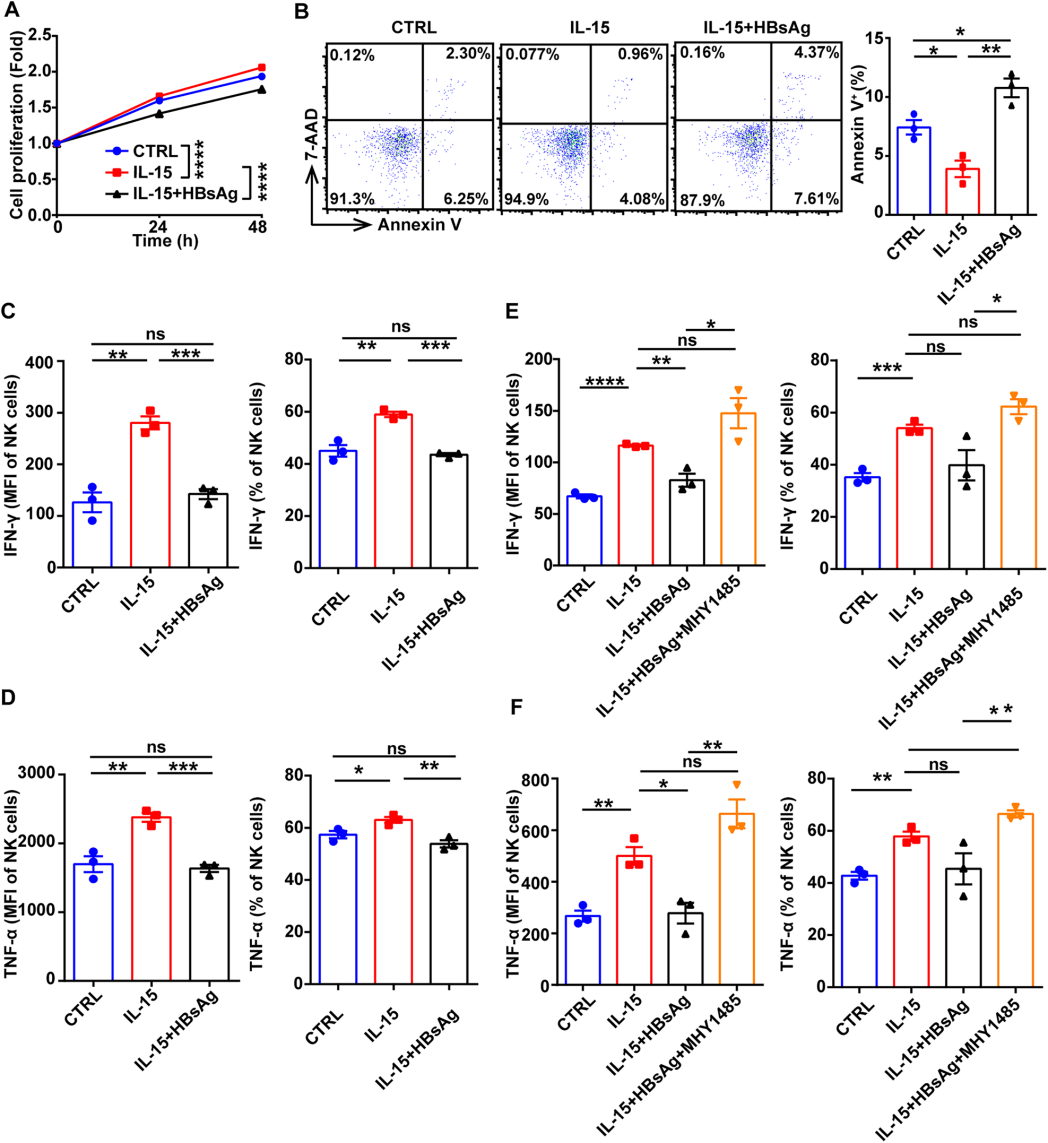
HBsAg disrupts the responsiveness of NK cells to IL-15 stimulation. **A**, **B** NK cells isolated from HDs were incubated with or without HBsAg for 24 h and 48 h in the presence of 20 ng/mL IL-15. The cell viability was measured with CCK8 (**A**), and the apoptosis rate was measured by flow cytometry with Annexin V/7AAD kit (**B**). **C**, **D** PBMCs from HDs were incubated with or without HBsAg for 48 h in the presence of 20 ng/mL IL-15, then the levels of IFN-γ (**C**) and TNF-α (**D**) in NK cells were measured by flow cytometry. **E**, **F** PBMCs from HDs were incubated with or without HBsAg for 48 h in the presence of 20 ng/mL IL-15, followed by the stimulation with 10 μM MHY1485 for 1 h, then the levels of IFN-γ (**E**) and TNF-α (**F**) in NK cells were measured by flow cytometry. Differences between these groups were analyzed using the two-way ANOVA for variables. Data were presented as mean ± SEM of at least three independent experiments. ns no significant. *p < 0.05, **p < 0.01, ***p < 0.001, ****p < 0.0001. SEM, standard error of the mean.

IL-15 is a multifunctional cytokine that activates NK cells through JAK/STAT and PI3K/AKT/mTOR pathways [33]. We found IL-15 could effectively stimulate the activation of mTOR pathway in a time-dependent manner, accompanied with the increase of downstream target gene HIF-1α (Fig. 3D). However, HBsAg significantly depressed IL-15-triggered mTOR signaling pathway and downregulated the expression of HIF-1α (Fig. 3E–G). Moreover, HBsAg diminished the promotive effects of IL-15 on the proliferation ability (Fig. 4A, B) and reversed the inhibitory effects of IL-15 on the apoptosis of NK cells. Meanwhile, HBsAg disrupted the production of IFN-γ and TNF-α of NK cells from HDs (Fig. 4C, D), which could be obviously reversed by mTOR agonist MHY1485 (Fig. 4E, F). Additionally, mTOR agonist MHY1485 could improve the uptake ability of 2-NBDG and the production of IFNγ and TNFα in NK cells from CHB patients (Fig. S3). These data suggest that HBsAg suppresses NK cell glycolysis via inhibiting IL-15-triggered mTOR signaling transduction.

HBeAg is a structural protein inside HBV that can be secreted into the blood and is one of the markers of HBV replication. Similar to HBsAg, we found that HBeAg also inhibited IL-15-activated mTOR signaling pathway in NK cells in a dose-dependent manner (Fig. S4A), and weakened the augmenting effects of IL-15 on the proliferation ability and function of NK-92 cells (Fig. S4B–E). To compare the influence of HBsAg and HBeAg on NK cells, NK-92 cells were pre-incubated with HBeAg and HBsAg, respectively. We observed both the pre-incubation with HBeAg and HBsAg could reduce the binding of HBeAg-APC to NK-92 cells, but the pre-incubation with HBsAg displayed a stronger effect than with HBeAg at the same concentrations (Fig. S4F). Therefore, we think that the binding ability of HBsAg to NK cells was stronger than that of HBeAg.

### HBsAg binds to IL-15Rβ on NK cells

**Fig. 5:**
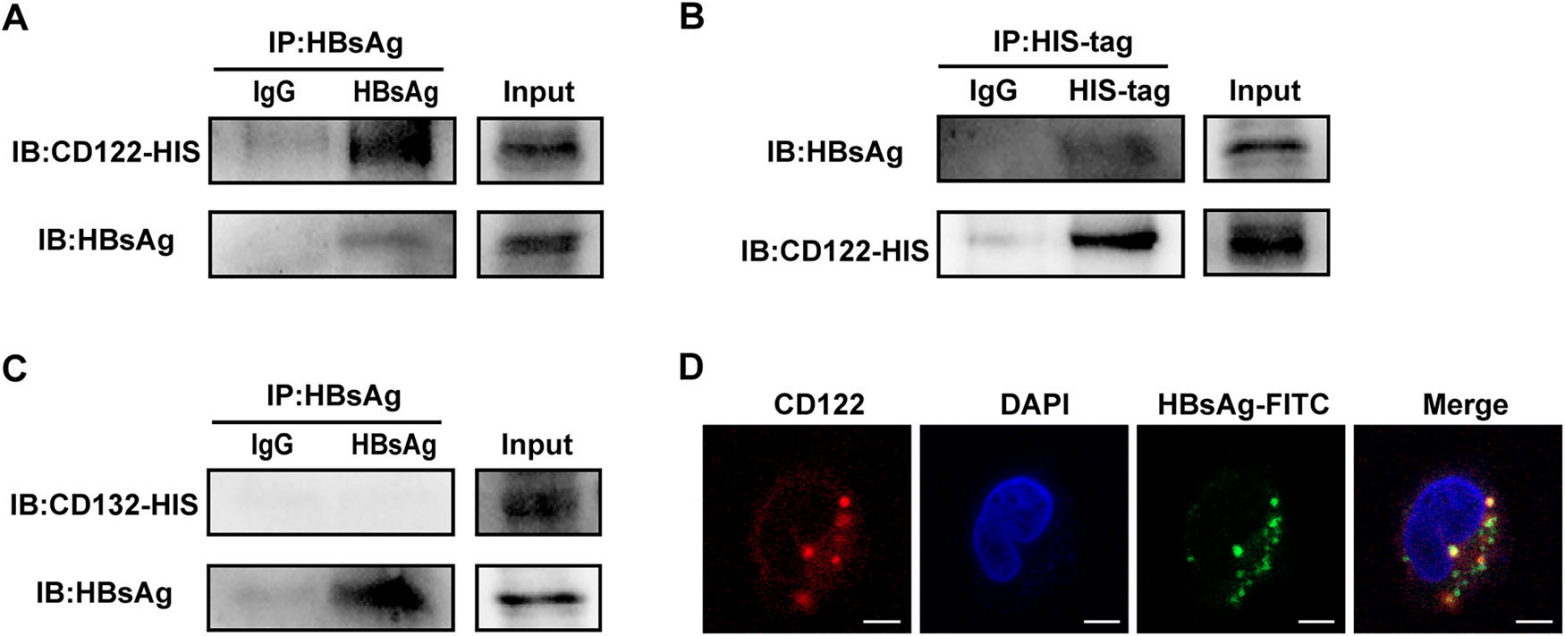
HBsAg binds to CD122 on NK cells. **A**–**C** HEK293T cells were transfected with pcDNA3.1-CD122-HIS (**A**, **B**) and pcDNA3.1-CD132-HIS (**C**) plasmid for 12 h, then incubated with 20 μg/mL HBsAg for 24 h. These cell lysates were collected for immunoprecipitation (IP) with indicated antibodies and IP complexes, followed by immunoblotting (IB) analysis. **D** Representative confocal microscopy images of NK-92 cells stained with CD122 (red) and DAPI (blue) after incubated with 20 μg/mL HBsAg-FITC (green) for 24 h. The scale bars are all 5 μm. One representative of three independent experiments.

The IL-15 receptor (IL-15R) complex consists of IL-15Rα (CD215), IL-15Rβ (CD122), and γ chain (CD132). Based the findings above, we speculated that HBsAg might competitively bind to the IL-15R on the surface of NK cells, thereby disturbing IL-15-triggered mTOR activity. To confirm this hypothesis, CD122 and CD132 were overexpressed in HEK293T cells, then treated with HBsAg for 24 h. Co-IP results showed that HBsAg could bind to CD122 (Fig. 5A, B), but didn’t bind to CD132 (Fig. 5C). We also confirmed the co-localization of HBsAg and CD122 on NK cells by immunofluorescence (Fig. 5D). These data indicate that HBsAg can competitively bind to CD122 on NK cells, blocking IL-15-triggered mTOR signaling and NK cell functions.

### Activation of mTOR in NK cells promotes viral clearance in HBV-carrier mice

**Fig. 6:**
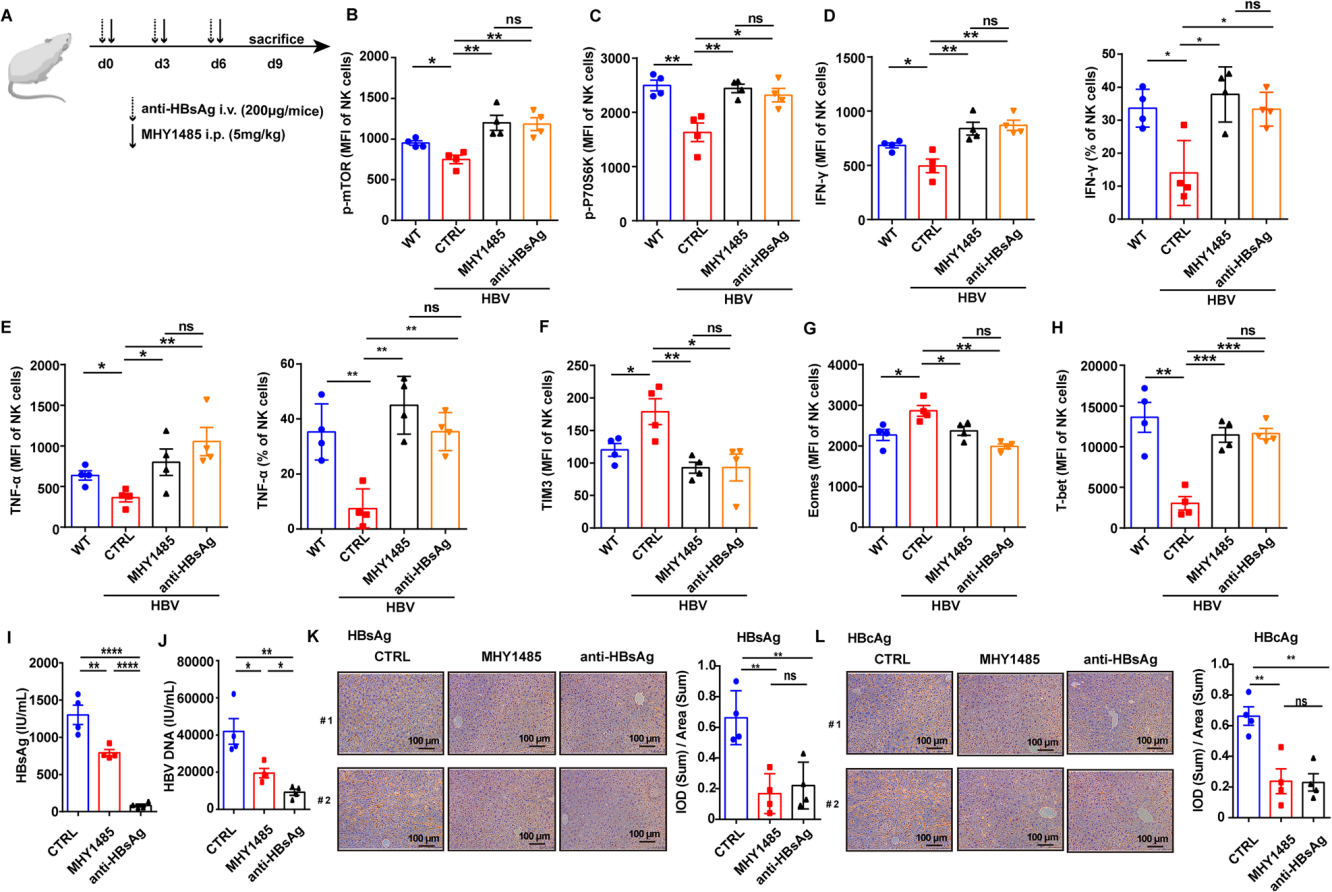
Activation of mTOR in NK cells promotes viral clearance in HBV-carrier mice. **A** The treatment schedule of HBV-carrier mice with HBsAg neutralizing antibody or mTOR agonist MHY1485. **B**–**H** Mononuclear cells of liver from WT and HBV-carrier mice were stimulated with 20 ng/mL murine IL-15 for 1 h, then MFI of *p*-mTOR (**B**), *p*-P70S6K (**C**), IFN-γ (**D**), TNF-α (**E**), TIM3 (**F**), Eomes (**G**), and T-bet (**H**) of NK cells was measured by flow cytometry. **I** Serum HBsAg levels were monitored by CLIA on day 9 post of the treatments. **J** Serum HBV DNA level was measured by RT-qPCR on day 9 post of the treatments. **K** IHC staining showing in situ HBsAg expression in hepatocytes, and HBsAg^+^ hepatocytes were quantified using Image-Pro Plus software. **L** IHC staining showing in situ HBcAg expression in hepatocytes, and HBcAg^+^ hepatocytes were quantified using Image-Pro Plus software. The scale bars are all 100 μm. *n* = 4. Differences between these groups were analyzed using the two-way ANOVA for variables. Data were presented as mean ± SEM. ns no significant. *p < 0.05, **p < 0.01, ***p < 0.001, ****p < 0.0001. SEM standard error of the mean.

To confirm the effects of mTOR-mediated NK cell function in vivo, HBV-carrier mouse model was prepared. NK cells isolated from liver and spleen of WT and HBV-carrier mice were performed RNA-seq. Consistent with NK cells of patients with CHB, many differentially expressed genes were found in NK cells from HBV-carrier mice (Fig. S5A), and the profile of differentially expressed genes involved in the glycolysis, such as GLUT1, HK2, PKM2, STAT3, and HIF1-α, showed similar changes between NK cells from HBV-carrier mice and those from CHB patients (Figs. 1A, B, and S5B). As shown in Fig. 6A, HBV-carrier mice were treated with mTOR agonist MHY1485 and HBsAg-neutralizing antibody, respectively. The results showed hepatic NK cells from HBV-carrier mice displayed low responsiveness to IL-15 stimulation compared to NK cells from WT mice, characterized as the depressed levels of *p*-mTOR and *p*-P70S6K. Notably, similar to the treatment of HBsAg neutralizing antibody, MHY1485 could restored the activation of mTOR signaling in response to IL-15 stimulation in NK cells from HBV-carrier mice (Figs. 6B, C and S6), accompanied with the upregulation of IFN-γ (Figs. 6D and S6) and TNF-α (Fig. 6E) and the downregulation of TIM3 (Fig. 6F). In addition, both MHY1485 and HBsAg neutralizing antibody could enhance the terminal differentiation of NK cells, showing decreased Eomes level and increased T-bet level (Fig. 6G, H). As expected, serum HBsAg and HBV DNA levels were decreased obviously in HBV-carrier mice treated with HBsAg-neutralizing antibody (Fig. 6I, J), as well as the expression of HBsAg and HBcAg in the liver tissues (Fig. 6K, L). Significantly, mTOR agonist MHY1485 exhibited similar roles to HBsAg neutralizing antibody (Figs. 6 and S6). These results suggest the activation of mTOR signaling augments NK cell function and HBV clearance.

### mTOR-activated NK cells enhance CD8^+^ T cell activity in HBV-carrier mice

**Fig. 7:**
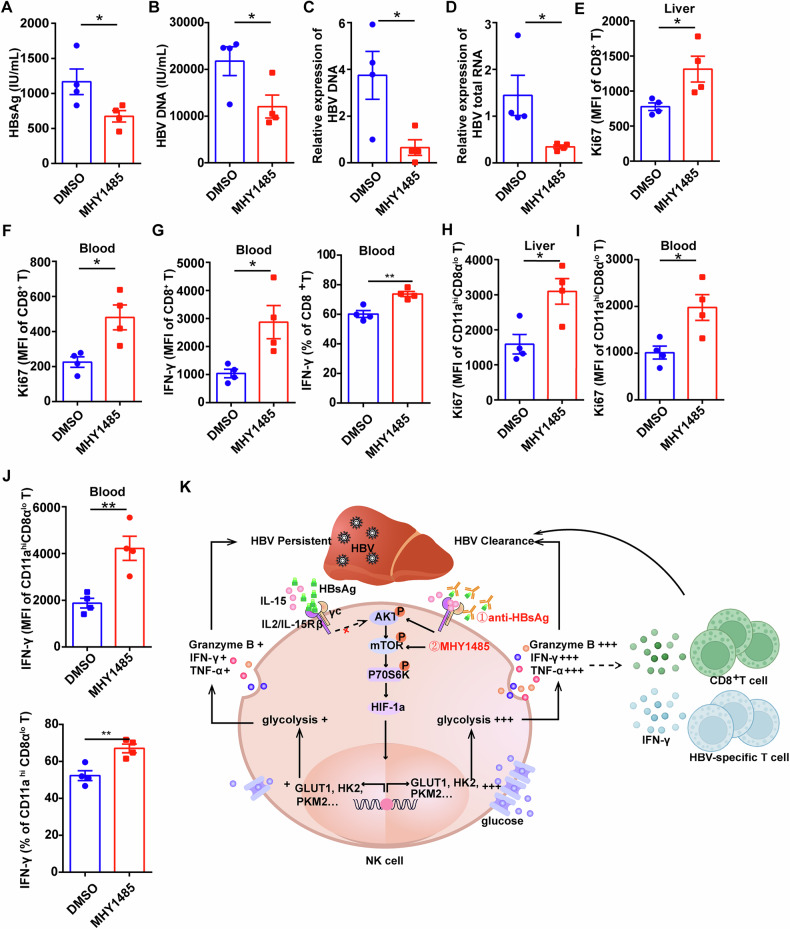
mTOR-activated NK cells enhance CD8^+^ T cell activity in HBV-carrier mice. NK cells isolated from liver and spleen of HBV-carrier mice were treated with 10 μM MHY1485 or DMSO for 4 h and then transferred to recipient HBV-carrier mice. The following analysis were performed on day 3 post of transferring. **A** Serum HBsAg level was detected by CLIA. **B** Serum HBV DNA level was measured by RT-qPCR. **C**, **D** Relative expression of HBV DNA (**C**) and HBV total RNA (**D**) in liver was measured by RT-qPCR. **E**–**G** The levels of Ki67 and IFN-γ in CD8^+^ T cells were measured by flow cytometry. **H**–**J** The levels of Ki67 and IFN-γ in CD11a^hi^CD8α^lo^ HBV-specific T cells were measured by flow cytometry. **K** Schematic model of HBsAg binding to the IL-15Rβ (CD122) on the surface of NK cells from patients with CHB, which blocks IL-15-triggered mTOR activity and reduces the expression of HIF-1α and downstream genes related to glycolysis, resulting in the dysfunction of NK cells and persistent HBV infection. HBsAg neutralizing antibody and mTOR agonist can restore mTOR signaling transduction, improving NK cell glycolysis and function and promoting HBV clearance. *n* = 4. Differences between these two groups were analyzed using the unpaired Student’s *t*-test for variables. Data were presented as mean ± SEM. *p < 0.05, **p < 0.01. SEM standard error of the mean.

To confirm the role of mTOR activation on NK cell-mediated anti-virus activity, NK cells isolated from HBV-carrier mice were treated with MHY1485 and then transferred into receipt HBV-carrier mice, which can avoid the influence of MHY1485 on other immune cells as previously reported [34, 35]. The results showed the levels of serum HBsAg and HBV DNA in mice transferred with MHY1485-treated NK cells were significantly reduced compared to that transferred with DMSO-treated NK cells (Figs. 7A, B and S7). Moreover, transferring MHY1485-treated NK cells also decreased the levels of HBV DNA and HBV total RNA in the liver tissues (Fig. 7C, D). Normally, besides recognizing and clearing virus-infected cells directly, NK cells also display regulatory role on other immune cells, especially CD8^+^ T cells. We found that MHY1485-treated NK cells increased the proliferation capacity (Fig. 7E, F) and IFN-γ secretion (Fig. 7G) of CD8^+^ T cells in HBV-carrier mice. Importantly, the expression of Ki67 and IFN-γ of the HBV-specific CD11a^hi^ CD8α^lo^ cells also increased by the transfer of MHY1485-treated NK cells (Fig. 7H–J). These data suggest that mTOR-activated NK cells can promote CD8^+^ T cells activity.

## Discussion

Chronic HBV infection is an immune-related liver disease. The interactions between the replication of virus and the anti-viral immunity of host determines the final result of HBV infection. As an important component of innate immune system, NK cells play vital roles in controlling virus by cytolysis activity and production of effector cytokines [36]. However, chronic HBV infection usually induces NK cell exhaustion, characterized as the phenotypic changes and dysfunctions. The most typical features of NK cells in patients with CHB include the upregulation of inhibitory receptors such as TIM-3, NKG2A, and Siglic-9, and the downregulation of activating receptors such as NKp30, NKG2D, and 2B4 [37–39]. In addition, NK cells in patients with CHB presented transcriptional similarity with exhausted T cells, such as the upregulation of transcription factors TOX and NR4A [28]. However, although the phenotype of NK cells has been integrally conflated with effector function, increasing studies in recent years have shown that the phenotype is not the only determinant for NK cell functions, the metabolic signals exert greater roles in NK cells.

Specific immune cells adopt metabolic pathways that support their functions. During cell development, proliferation, and activation, cells will undergo metabolic reprogramming. As effectors of innate immune responses, NK cells have characteristic metabolic configurations that facilitate their functions. Resting NK cells have low basal metabolic rates. Upon stimulation, the rates of glucose-driven glycolysis and OXPHOS are increased in NK cells [11], which is essential for NK cells to kill target cells and produce cytokines. In this study, we found that the glycolysis capacity of NK cells in PBMCs and liver from patients with CHB was significantly decreased, which was correlated with the impaired activation of NK cells. Similar to HBV-specific T cells, NK cells of patients with CHB exhibited mitochondrial damage. However, the genes associated with lipid metabolism and amino acid metabolism in NK cells of CHB patients did not show significant change compared to HDs (data not shown).

mTOR is well defined as a positive regulator of various metabolic processes in diverse cell types. mTOR activity is low in resting NK cells, but powerfully enhanced by cytokine stimulation in both human and mouse NK cells, accompanied by highly increased metabolic rates [26]. Donnelly et al. showed mTOR activity is necessary for glycolytic reprogramming, and this metabolic shift is required for NK cell to produce IFN-γ and granzyme B [29]. In addition, mTOR can promote protein synthesis by regulating the downstream transcription factors such as HIF-1α and SREBP. Here, we found the transduction of mTOR pathway in NK cells from patients with CHB and HBV-carrier mice was inhibited, and accompanied with the changes of gene profile related to glycolysis. Studies have shown mTOR is important for NK cell development and maturation. mTORC1 and mTORC2 promote the development of NK cells by inducing E4BP4 and T-bet, respectively; but, the deletion of mTORC1 and mTORC2 can reduce the proportion of mature CD27^−^CD11b^+^ and KLRG1^+^ NK cells in mice [40, 41]. Similarly, we observed the activation of mTOR in NK cells was suppressed in HBV-carrier mice, accompanied with the increased Eomes and the decreased T-bet, indicating the disruption of mTOR signaling pathway blocks the terminal differentiation of NK cells.

IL-15 is a pleiotropic cytokine that is essential for NK cell survival, maturation, and function by activating Ras-Raf-MAPK, JAK/STAT, and PI3K/AKT/mTOR pathways [42]. Previous studies showed the level of phosphorylated-STAT5 (p-STAT5), a non-mTOR dependent pathway, in NK cells of patients with CHB did not show significant change compared to HDs and nor could be induced by IL-15 stimulation, which further confirmed chronic HBV infection impaired the glycolysis and function of NK cells via mTOR signaling [26, 28]. The level of IL-15 is very low under homeostasis, but is upregulated during inflammation and infection. In HBV-carrier mice, intrahepatic overexpression of IL-15 inhibits HBV replication in an IFN-β-dependent manner [43]. Under telbivudine treatment, CD56^bright^ NK cells exhibit strong anti-HBV function in an IL-15-dependent manner [44]. Additionally, IL-15 can play anti-viral roles by synergistically enhancing the function of specific CD8^+^ T cells with IFN-α [45]. Our previous study showed that HBsAg and HBeAg significantly suppressed the production of IFN-γ and TNF-α in NK-92 cells, and the combination of them (at the same dosage) exacerbated this effect [46]. In this study, we found HBsAg could inhibit the responsiveness of NK cells to IL-15 and impair the activation of mTOR in NK cells, resulting in the decreased expression of glycolytic-related proteins and glucose metabolism. Although HBeAg exhibited similar suppressive effect on mTOR, the clinical data showed the serum HBeAg load of patients with CHB was significantly lower than that of HBsAg [2], and the binding ability of HBsAg to NK cells was significantly stronger than that of HBeAg. Therefore, we subsequently focused on the interference of HBsAg on NK cells.

Unlike other γ chain cytokines, IL-15 must be trans-delivered by IL-15Rα to IL-15Rβ (CD122)/γ (CD132) heterodimers on NK cell surface to exert effects [47]. As a shared receptor for IL-2 and IL-15, CD122 is one of the earliest markers of NK cell development. In the bone marrow, the common lymphoid progenitors develop into the NK progenitors (NKP) that express high level of CD122 to maintain the responsiveness to IL-15 and promote NK cells maturation, homeostasis, and function [48]. Han et al. showed the level of CD122 on T and NK cells was reduced and displayed an obvious negative correlation with the levels of HBsAg and HBV DNA [49], which might explain why the elevated IL-15 could not exert positive effects on NK cells in patients with CHB. We also observed downregulated expression of CD122 in NK cells of patients with CHB, but HBsAg did not directly affect the expression of CD122 (data not shown). Significantly, we confirmed HBsAg could competitively bind with CD122, impeding the responsiveness of NK cells to IL-15 and IL-15-triggered signaling transduction.

Currently, IL-15 agonists and recombinant IL-15 protein alone or in combination with immunocheckpoint inhibitors have shown significant anti-tumor effects in preclinical trials [50]. Huang et al. found knocking down cytokine-inducible SH2-containing protein (CIS) could relieve the suppression of IL-15 signaling and activate PI3K/AKT/mTOR pathway of NK cells, which upregulated the glycolysis and OXPHOS rates, thus promoting the effector function of NK cells [51, 52]. However, due to the presence of HBsAg that binds to CD122, these approaches may be ineffective for improving the responsiveness of NK cells to IL-15 in patients with CHB. Notably, we found that HBsAg-neutralizing antibody could restore the response of NK cells to IL-15, activate the mTOR pathway, and enhance the effector function of NK cells. However, Valeria et al. showed that HBsAg clearance had little effect on the immune response of CD8^+^ T cells [53]. These findings indicate that HBsAg-mediated the immunosuppressive effect on NK cells was far greater than that on CD8^+^ T cells. Unfortunately, there is no efficient way in clinical to achieve HBsAg seroconversion at present. mTOR agonist MHY1485 can activate mTOR pathway by targeting ATP domain. In this study, we found MHY1485 could restore the IL-15/mTOR signaling in NK cells of HBV-carrier mice, and the transferring of NK cells treated with MHY1485 could display a powerful anti-viral function directly and simultaneously enhanced the activity of CD8^+^ T cells. Therefore, mTOR agonists are expected to be potential candidates for improving NK cell function in patients with CHB.

Except the NK cells, HBsAg and HBeAg also affect the function of other immune cells in different manner, such as CD8^+^T cells and dendritic cells (DCs). For example, HBeAg induced the conversion of bone marrow-derived DCs into regulatory DCs, inhibiting T cell activation [54]; HBsAg abrogated TLR9-triggered maturation of plasmacytoid DCs, resulting in the reduction of pro-inflammatory cytokines, such as IL-12, IFN-α, and IFN-λ1 [55]. Moreover, our previous study confirmed HBsAg, but not HBeAg, was associated with the reduction of free cholesterol levels and the impaired lipid raft formation on DCs [56]. However, our study might have certain limitations, such as lacking further characterizing CHB disease phases in CHB patients. So, it is difficult to understand whether chronic HBV infection result the similar effects on IL-15-triggered mTOR activity in NK cells from patients with CHB at different disease stages, such as immune-tolerant CHB, immune-active CHB, and inactive CHB. In addition, NK cells in liver possess circulating conventional NK cells (cNKs) and liver-resident NK cells (LrNK). cNKs circulate through the liver and vasculature, while LrNKs are believed to remain in the liver. Considering cNK cells and LrNKs display different functions and transcription factor profiles, it is meaningful to clarify the characteristics of different hepatic NK cell subsets during chronic HBV infection in the future, such as using human-liver-chimeric mice.

In summary, HBsAg binds to the IL-15Rβ (CD122) on the surface of NK cells of patients with CHB, which blocks IL-15-triggered mTOR activity and reduces the expression of HIF-1α and downstream genes related to glycolysis (Fig. 7K). So, chronic HBV infection disrupts glucose metabolism in NK cells, resulting in the impairments of cytokine production and anti-HBV effects. HBsAg neutralizing antibody and mTOR agonist can restore mTOR signaling transduction, improving NK cell function and promoting HBV clearance. This study revealed a new mechanism by which chronic HBV infection induced NK cell dysfunction, indicating activating mTOR in NK cells might be an efficient therapeutic strategy for patients with CHB. The development of mTOR agonists will provide new options and benefits for patients with CHB, especially for those immunocompromised patients.

## Supplementary information

Supplementary Materials-Chronic HBV infection impairs the glucose metabolism and effector function of NK cells via HBsAg/IL-15/mTOR axis

Full and uncropped western blots

Reproducibility checklist

## Data Availability

The data presented in this study are available upon reasonable request from the corresponding author. The published scRNA-seq dataset that support the findings of this study is available through managed access via the Gene Expression Omnibus (GSE182159).
