# Supplementary material for: Chronic HBV infection impairs the glucose metabolism and effector function of NK cells via HBsAg/IL-15/mTOR axis
Source: Cell Death Dis. 2025 Oct 13;16(1):721. doi: 10.1038/s41419-025-08069-y (PMC12518832; doi:10.1038/s41419-025-08069-y)
Supplement: Supplementary file 1 — Supplementary Materials-Chronic HBV infection impairs the glucose metabolism and effector function of NK cells via HBsAg/IL-15/mTOR axis [file 41419_2025_8069_MOESM1_ESM.pdf]

Supplementary Information for

Chronic HBV infection impairs the glucose metabolism and effector function of NK cells via HBsAg/IL-15/mTOR axis

Yating Yu[1], Zixuan Wang[1], Ailu Yang[1], Yucan Wang[1], Cuiping Bao[1], Li Zhuo[2], Qiuju Han[1], Huajun Zhao[1]*, Jian Zhang[1]*

[1] State Key Laboratory of Discovery and Utilization of Functional Components in Traditional Chinese Medicine, Institute of Immunopharmaceutical Sciences, School of Pharmaceutical Sciences, Shandong University, Jinan, Shandong, 250012, China

[2] State Key Laboratory of Microbial Technology, Institute of Microbial Technology, Shandong University, Qingdao, Shandong, China

*** Corresponding authors. Address:** State Key Laboratory of Discovery and Utilization of Functional Components in Traditional Chinese Medicine, Institute of Immunopharmaceutical Sciences, School of Pharmaceutical Sciences, Shandong University, No. 44 Wenhua West Road, Jinan, Shandong, 250012, China.

E-mail address: Jian Zhang, zhangj65@sdu.edu.cn; Huajun Zhao, zhaohuajun89@sdu.edu.cn.

Supplementary materials and methods

Glucose uptake assessment

Glucose uptake was measured using 2-(N-(7-Nitrobenz-2-oxa-1,3-diazol-4-yl)Amino)-2-Deoxyglucose (2-NBDG, APExBIO, USA). Cells were resuspended in RPMI-1640 medium supplemented with 10% FBS in the presence of 10 μM 2-NBDG for 15 min at 37°C, and then the surface markers were stained by indicated fluorescently labeled antibodies for 30 min and analyzed with FlowJo software (FlowJo, USA).

ECAR analysis

Purified primary NK cells were stimulated with 500 U/mL rhIL-2 at 37°C in a 5% CO_2 incubator for 18 h, and these cells were then cultured with Seahorse XF DMEM medium (pH 7.4, Agilent, USA) containing 2 mM L-Glutamine, and placed in a CO2-free incubator for 1 h. During this period, 10 mM glucose (Sigma, USA), 1 μM oligomycin (MCE, USA) and 50 mM 2-DG (Sigma, USA) were added. Then the ECAR of NK cells was measured using the Seahorse XFe24 Analyzer (Agilent, USA).

HBsAg, HBeAg and HBV DNA detection

Specific chemiluminescent immunoassay assay (CLIA) kits were used to analyze the serum levels of HBsAg and HBeAg (Autobio, Zhengzhou, China) according to the manufacturer's instructions. Serum HBV DNA levels were detected by RT-qPCR using the specific HBV nucleic acid assay kit (Sansure Biotech, Changsha, China).

Glucose and lactate contents detection

Glucose and lactate contents were detected by liquid samples glucose assay kit (Applygen, Beijing, China) and lactate assay kit (Nanjing Jiancheng Bioengineering Institute, Nanjing, China), respectively.

Immunoprecipitation (IP)

pcDNA3.1-CD122-HIS and pcDNA3.1-CD132-HIS vectors were transfected into HEK293T cells for 12 h, then 200 μg HBsAg was added and incubated for 24 h. Cells were lysed by 0.5% NP-40 containing protease inhibitor cocktail and phosphatase inhibitor cocktail, and then broken by ultrasonic. These cell lysates were centrifuged

at 12000 × g for 10 min at 4°C and the supernatants were collected. The antibodies were incubated with protein A/G Magnetic Beads (Bimake, USA) for 1 h at room temperature, followed by incubating with these cell lysates overnight at 4°C. The beads were then washed with 300 mM NaCl and 150 mM NaCl (configured with 3M NaCl and 0.05% NP-40), respectively, followed by immunoblotting (IB) analysis.

Confocal microscopy

Primary NK cells or NK-92 cells were treated with 20 μg/mL FITC-labeled HBsAg (HBsAg-FITC) for 24 h, followed by incubation with 5 μM membrane fluorescent probe DiD (Beyotime, Shanghai, China) or anti-human CD122 antibody (Biolegend, USA) at 37°C for 30 min in the dark. For mitochondrial membrane potential detection, cells were stained with the Mito-Tracker Red CMXRos (Beyotime, Shanghai, China) at 37°C for 30 min in the dark. These cells were then washed with 1× PBS and sealed with antifade mounting medium with DAPI (Beyotime, Shanghai, China). Images were captured using a confocal laser-scanning microscope (Carl Zeiss AG, Jena, Germany).

Reactive oxygen species (ROS) detection

Cells were stained with the ROS assay kit (Beyotime, Shanghai, China) at 37°C for 20 min in the dark and then washed with 1× PBS. Data were collected using a FACSCelesta system (BD Biosciences, USA) and analyzed with FlowJo software (FlowJo, USA).

Immunohistochemistry (IHC)

HBsAg and HBcAg in liver tissues were stained with anti-HBsAg and anti-HBcAg monoclonal antibody (dilution: 1:500, Gene Tech, Shanghai, China) respectively, and then incubated with horseradish peroxidase-conjugated goat anti-mouse IgG (ZSGB Bio, Beijing, China).

RNA-seq analysis

NK cells were isolated from HDs and patients with CHB, and WT and HBV-carrier mice, and total RNA of these NK cells were was extracted by TRIzol reagent. Sequencing libraries were generated using the TruSeq RNA sample preparation kit (Illumina, San Diego, CA, USA) and sequenced on the Illumina Novaseq™ 6000

(LC-Bio Technology CO., Ltd., Hangzhou, China) following the vendor's recommended protocol. Briefly, A cDNA library constructed by technology from the pooled RNA from < sample description > samples of <research species > was sequenced run with Illumina NovaseqTM 6000 sequence platform. Thus, to get high quality clean reads, reads were further filtered by Cutadapt (https://cutadapt.readthedocs.io/en/stable/, version:cutadapt-1.9). Then, we aligned reads of all samples to the < research species > reference genome using HISAT2 (https://daehwankimlab.github.io/hisat2/, version:hisat2-2.2.1) package. These mapped reads of each sample were assembled using StringTie (http://ccb.jhu.edu/software/stringtie/, version:stringtie-2.1.6) with default parameters. Genes differential expression analysis was performed by DESeq2 software between two different groups (and by edgeR between two samples). We use R to perform correlation analysis, and principal component analysis (PCA) was performed with princomp function of R (http://www.r-project.org/) in this experience. And, differentially expressed genes were then subjected to enrichment analysis of GO functions and KEGG pathways using software GSEA (v4.1.0) and MSigDB.

Supplementary Table 1. Characteristics of CHB patients and healthy donors.

Variables	HDs	HBeAg^{+} CHB	HBeAg^{-} CHB
Number	165	170	150
Age, year	51 (19~76)	43 (23~70)	47 (24~74)
Gender, male(%)	71 (43.0%)	118 (69.4%)	87 (58.0%)
HBsAg (IU/mL)	/	3657.9 (523.8~11132.2)	2262.2 (558.6~33267.8)
HBeAg (IU/mL)	/	44.2 (1.5~1789.4)	0.4 (0.3~0.9)

The inclusion criteria for HDs (aged 19 to 76 years old) were: without liver disease of HBV and other etiologies (HCV, hepatitis D virus (HDV), alcoholic liver disease, nonalcoholic fatty liver disease, primary biliary cholangitis, autoimmune hepatitis, hereditary metabolic liver disease, decompensated cirrhosis, major systematic diseases, HCC or other malignancies. The inclusion criteria for HBeAg-positive CHB patients (aged 23 to 70 years old) and HBeAg-negative CHB patients (aged 24 to 74 years old) were : HBV DNA > 2×10^4 IU/ml without prior antiviral therapy; without liver disease of other etiologies (HCV, hepatitis D virus (HDV), alcoholic liver disease, nonalcoholic fatty liver disease, primary biliary cholangitis, autoimmune hepatitis, hereditary metabolic liver disease, decompensated cirrhosis, major systematic diseases, HCC or other malignancies. Data were presented by median in non-normally distribution. HBsAg, hepatitits B surface antigen; HBeAg, hepatitits B e antigen.

Supplementary Table 2. Antibodies used for Flow Cytometry.

Antibodies	Supplier	Cat No.
FITC anti-human CD3	BioLegend	300306
APC/Cy7 anti-human CD56	BioLegend	318332
Alexa Fluor 700 anti-human CD56 (NCAM)	BioLegend	362522
PE anti-human CD107a	BioLegend	328608
Alexa Fluor 647 anti-human/mouse Granzyme B	BioLegend	515406
PE anti-human IFN-γ	BioLegend	502509
PE/Dazzle 594 anti-human TNF-α	BioLegend	502946
Phospho-mTOR(Ser2448) Antibody, eFluor 450	Invitrogen	48-9718-42
Phospho-mTOR (Ser2448) Antibody , PE	Invitrogen	12-9718-42
Phospho-S6 (Ser235, Ser236) Antibody , PE	Invitrogen	12-9007-42
Phospho-S6 (Ser235, Ser236) Antibody, eFluor 450	Invitrogen	48-9007-42
PerCP/Cyanine5.5 anti-human CD122 Antibody	BioLgend	339012
eFluor 506 Fixable Viability Dye (Mouse/human)	Invitrogen	65-0866-14
APC/Cyanine7 anti-mouse NK-1.1 Antibody	BioLegend	108724
FITC anti-mouse CD3 Antibody	BioLegend	100204
EOMES Monoclonal Antibody, PE-eFluor 610	Invitrogen	61-4875-82
PE/Cyanine7 anti-T-bet Antibody	BioLegend	644824
PE-Cy7 Hamster Anti-Mouse CD3e	BD Pharmingen	552774
CD8a Monoclonal Antibody, FITC	Invitrogen	11-0081-85
Ki-67 Monoclonal Antibody, eFluor 450	Invitrogen	48-5698-82
CD8a Monoclonal Antibody, PE	Invitrogen	12-0081-83
PerCP/Cyanine5.5 anti-mouse CD11a Antibody	BioLegend	101124
BV711 Rat Anti-Mouse IFN-γ	BD Pharmingen	564336
TNF alpha Monoclonal Antibody, PE	Invitrogen	12-7321-82
Brilliant Violet 785 anti-mouse Tim-3 Antibody	BioLegend	119725

Supplementary Table 3. The RT-qPCR primers sequences.

Primers	Sequences (5'-3')
Human-GAPDH-F	GTCTCCTCTGACTTCAACAGCG
Human-GAPDH-R	ACCACCCTGTTGCTGTAGCCAA
Human-GLUT1-F	TTGCAGGCTTCTCCAACTGGAC
Human-GLUT1-R	CAGAACCAGGAGCACAGTGAAG
Human-HK2-F	GAGTTTGACCTGGATGTGGTTGC
Human-HK2-R	CCTCCATGTAGCAGGCATTGCT
Human-PKM2-F	ATGGCTGACACATTCCTGGAGC
Human-PKM2-R	CCTTCAACGTCTCCACTGATCG
Human-LDHA-F	GGATCTCCAACATGGCAGCCTT
Human-LDHA-R	AGACGGCTTTCTCCCTCTTGCT
Human-STAT3-F	CTTTGAGACCGAGGTGTATCACC
Human-STAT3-R	GGTCAGCATGTTGTACCACAGG
Human-HIF-1α-F	TATGAGCCAGAAGAACTTTTAGGC
Human-HIF-1α-R	CACCTCTTTTGGCAAGCATCCTG
Mouse-GAPDH-F	AGGTCGGTGTGAACGGATTTG
Mouse-GAPDH-R	TGTAGACCATGTAGTTGAGGTCA
HBV-DNA-F	CACAT AGGATTCCTAGGACC
HBV-DNA-R	GGTGAGTGATTGGAGGTTG
HBV-total RNA-F	TCACCAGCACCATGCAAC
HBV-total RNA-R	AAGCCACCCAAGGCACAG

Supplementary Table 4. Antibodies used for western blotting.

Antibodies	Supplier	Cat No.
Phospho-AKT-S473 Rabbit pAb	ABclonal	AP0098
AKT Rabbit mAb	ABclonal	A17909
Phospho-mTOR (Ser2448) Rabbit mAb	Cell Signaling Technology	5536
mTOR Rabbit pAb	ABclonal	A2445
Phospho-P70S6K (Thr389) Rabbit mAb	Cell Signaling Technology	9234
P70S6K Polyclonal antibody	Proteintech	14485-1-AP
β-Actin Rabbit mAb	ABclonal	AC026
Mouse anti His-Tag mAb	ABclonal	(AE003)
Hepatitis B Virus Surface Monoclonal Antibody	Invitrogen	MA1-7603
HRP Goat Anti-Rabbit IgG (H+L)	ABclonal	AS014
Goat Anti-mouse IgG HRP	Abmart	M21005
Mouse IgG	Millipore	CS200621

SUPPLEMENTARY FIGURE

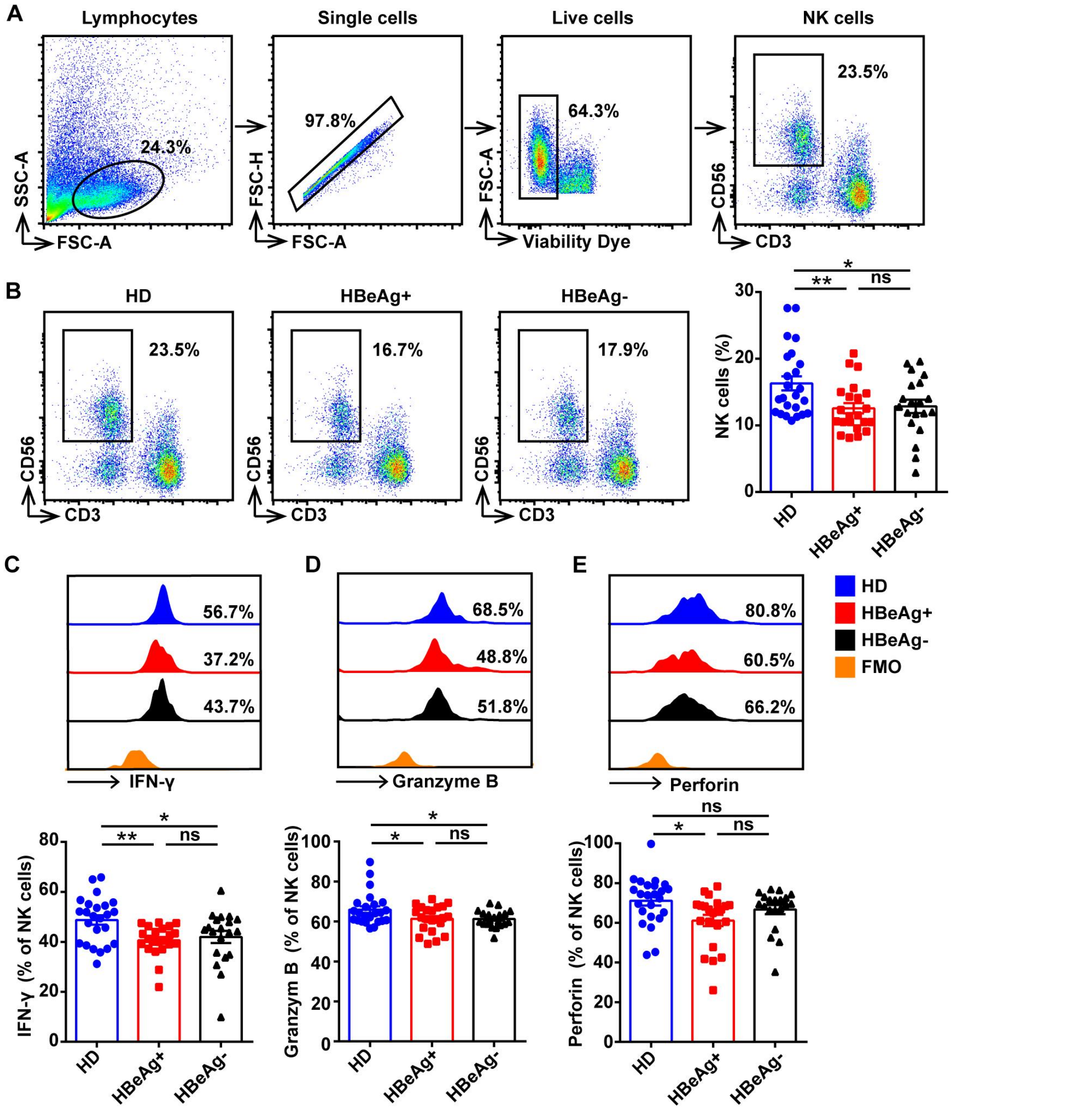

Supplementary Figure 1. The frequency and function of NK cells are affected by chronic HBV infection. (A) Gating strategy for flow cytometry analysis of NK cells. (B) The percentage of NK cells in HDs (n=24), HBeAg^{+} (n=22) and HBeAg^{-} (n=20) patients with CHB was measured by flow cytometry. (C-E) PBMCs from HDs and patients with CHB were treated with PMA (30 ng/mL) and ionomycin (1 μg/mL) for 4 h, then the levels of IFN-γ (C), granzyme B (D) and perforin (E) in NK cells were analyzed by flow cytometry. Differences between these groups were analyzed using the two-way ANOVA for variables. Data were presented as mean ± SEM. ns, no significant. *$p < 0.05$, **$p < 0.01$, ***$p < 0.001$, ****$p < 0.0001$. SEM, standard error of the mean.

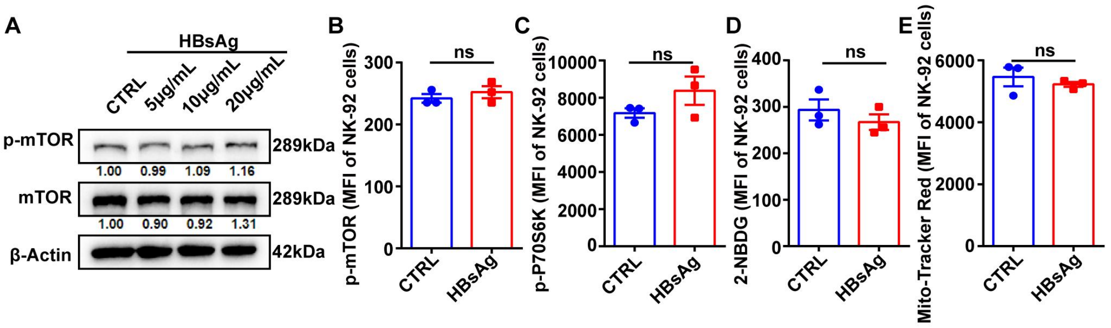

Supplementary Figure 2. HBsAg doesn't directly affect the activity of mTOR pathway of NK cells. (A) NK-92 cells were incubated with 5, 10 and 20 μg/mL HBsAg for 24 h, then the indicated molecules were analyzed by western blotting. Band intensities were quantified using ImageJ software and normalized to CTRL group. (B-C) NK-92 cells were incubated with 20 μg/mL HBsAg for 24 h, then MFI of *p*-mTOR (B) and *p*-P70S6K (C) was measured by flow cytometry. (D-E) NK-92 cells were incubated with 20 μg/mL HBsAg for 24 h, then MFI of 2-NBDG (D) and Mito-Tracker Red (E) was measured by flow cytometry. Differences between these two groups were analyzed using the unpaired Student's *t* test for variables. Data were presented as mean ± SEM. ns, no significant. SEM, standard error of the mean.

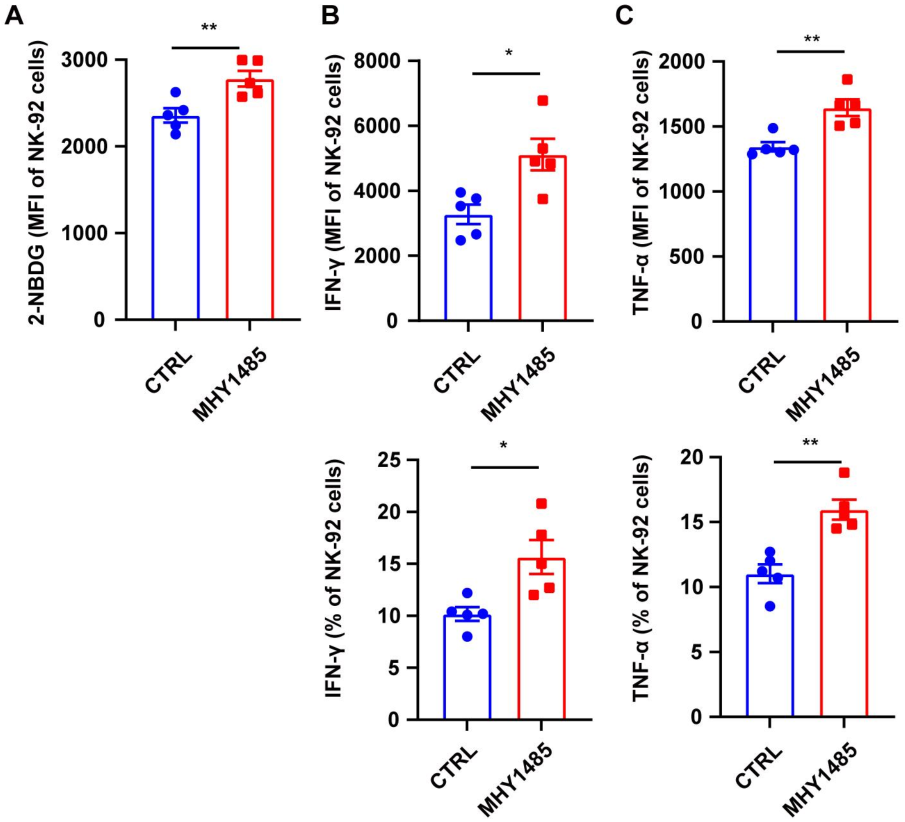

Supplementary Figure 3. mTOR agonist MHY1485 reversed the dysfunctional NK cells from CHB patients. NK cells from HBeAg$^-$ patients with CHB were stimulated with 10 μM MHY1485 for 1 h, then the levels of 2-NBDG (A), IFN-γ (B) and TNF-α (C) of NK cells were measured by flow cytometry. Differences between these two groups were analyzed using the unpaired Student's t test for variables. Data were presented as mean ± SEM of at least three independent experiments. *$p < 0.05$, **$p < 0.01$, ***$p < 0.001$, ****$p < 0.0001$. SEM, standard error of the mean.

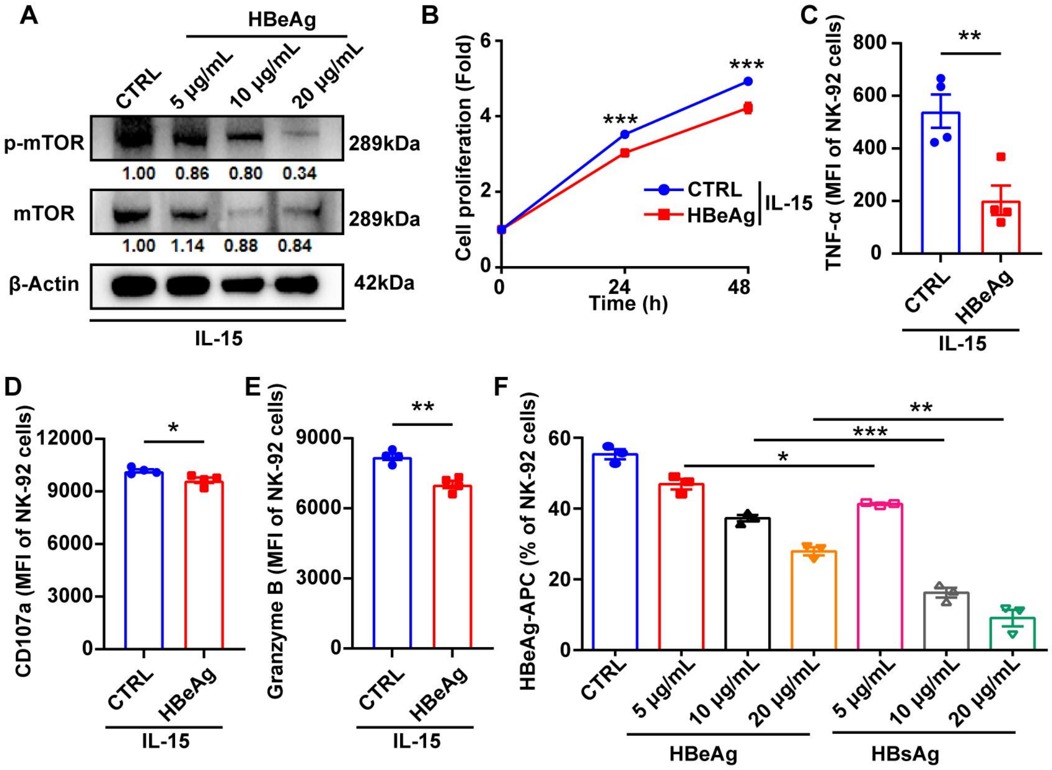

Supplementary Figure 4. HBeAg inhibits IL-15-induced mTOR pathway and effector function of NK cells. (A) NK-92 cells were incubated with 5, 10, 20 μg/mL HBeAg for 24 h and then stimulated with 20 ng/mL IL-15 for 1 h, the indicated molecules were analyzed by western blotting. Band intensities were quantified using ImageJ software and normalized to CTRL group. (B) NK-92 cells were incubated with or without HBeAg for 24 and 48 h in the presence of 20 ng/mL IL-15, then the cell viability was measured with CCK8. (C-E) NK-92 cells were incubated with or without HBeAg for 24 h in the presence of 20 ng/mL IL-15, then the expression levels of TNF-α (C), CD107a (D) and granzyme B (E) were analyzed by flow cytometry. (F) NK-92 cells were incubated with 5, 10, 20 μg/mL HBeAg and HBsAg for 2 h respectively, and then incubated with 10 μg/mL APC-labeled HBeAg (HBeAg-APC) for 2 h. The percentage of APC^+ NK-92 cells were analyzed by flow cytometry. Differences between two groups were analyzed using the unpaired Student's *t* test for variables, and differences among multiple groups were analyzed using the two-way ANOVA for variables. Data were presented as mean ± SEM. *$p < 0.05$, **$p < 0.01$, ***$p < 0.001$. SEM, standard error of the mean.

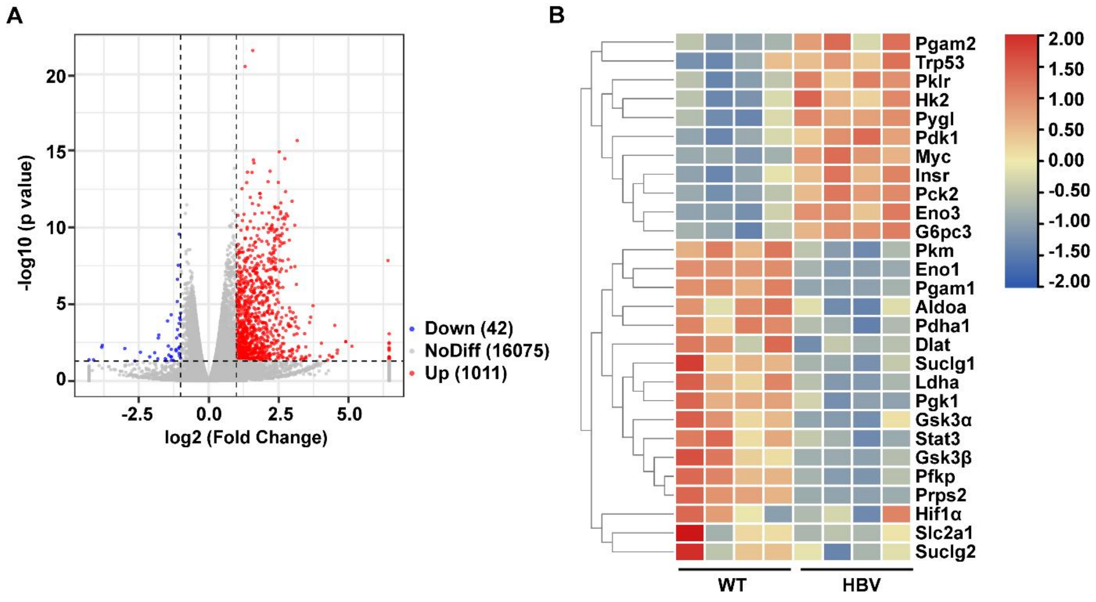

Supplementary Figure 5. Genes involved in the glycolysis of NK cells are downregulated in HBV-carrier mice. (A) Hepatic and splenic NK cells were isolated from WT and HBV-carrier mice for RNA-seq analysis, volcano plot showed differentially expressed mRNAs. (B) Heatmap analysis showed differentially expressed genes associated with the glycolysis of NK cells from WT and HBV-carrier mice.

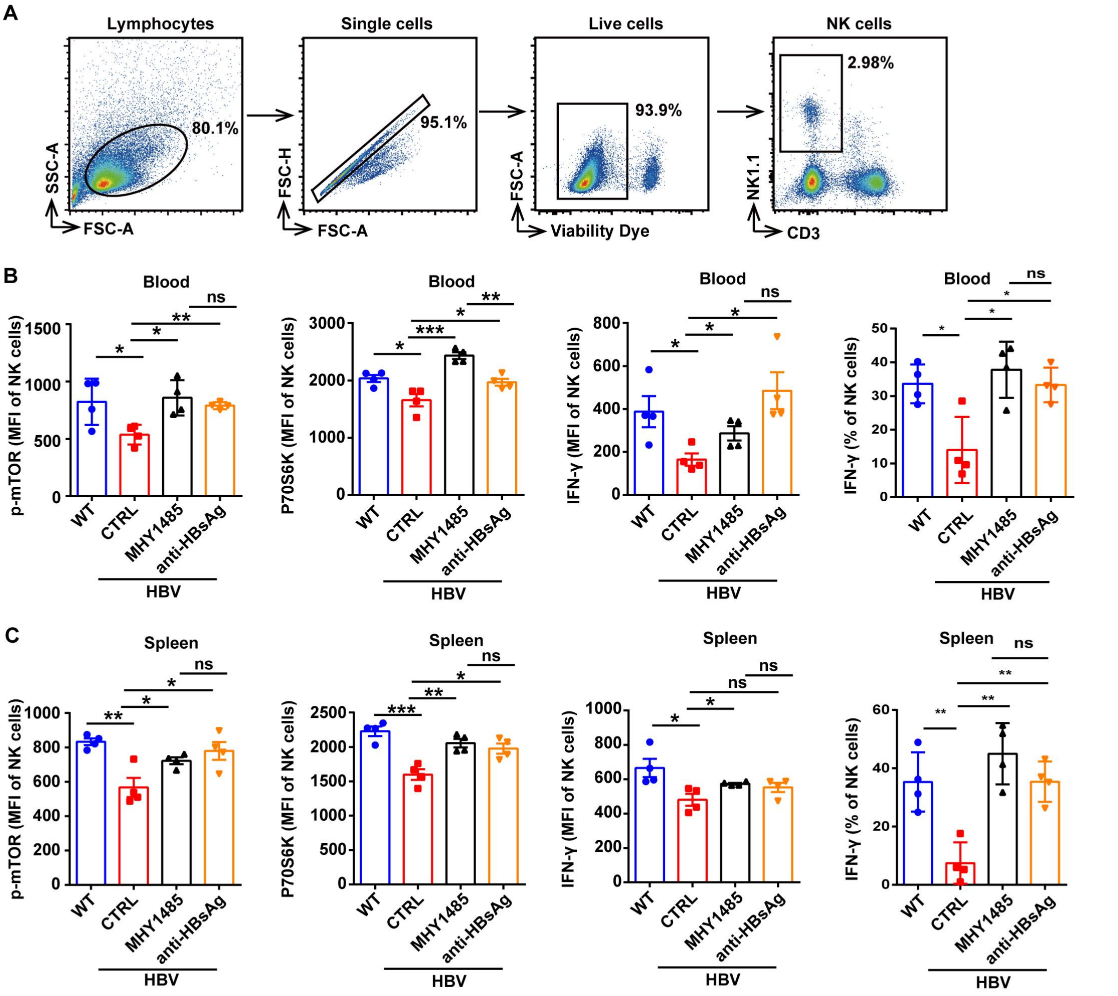

Supplementary Figure 6. The activation of mTOR promotes NK cell function in blood and spleen of HBV-carrier mice. HBV-carrier mice were treated with HBsAg neutralizing antibody or mTOR agonist MHY1485. (A) Gating strategy for flow cytometry analysis of NK cells. (B-C) Mononuclear cells of peripheral blood and spleen from WT and HBV-carrier mice were stimulated with 20 ng/mL murine IL-15 for 1 h, then MFI of *p*-mTOR, *p*-P70S6K and IFN-γ of NK cells in blood (B) and spleen (C) was measured by flow cytometry. n=4. Differences between these groups were analyzed using the two-way ANOVA for variables. Data were presented as mean ± SEM. ns, no significant. *$p < 0.05$, **$p < 0.01$, ***$p < 0.001$. SEM, standard error of the mean.

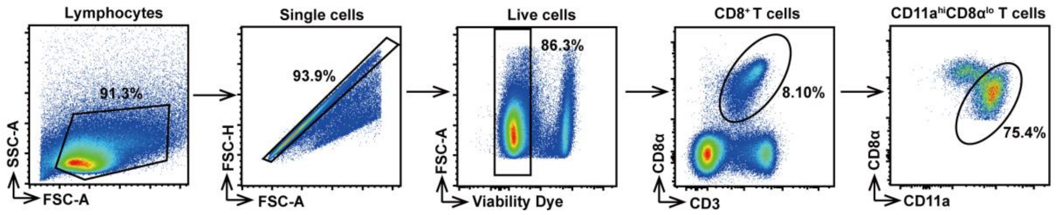

Supplementary Figure 7. Gating strategy of flow cytometry analysis of CD8^{+} T cells and CD11a^{hi} CD8α^{lo} HBV-specific T cells.
