# Supplementary material for: Chronic HBV infection impairs the glucose metabolism and effector function of NK cells via HBsAg/IL-15/mTOR axis
Source: Cell Death Dis. 2025 Oct 13;16(1):721. doi: 10.1038/s41419-025-08069-y (PMC12518832; doi:10.1038/s41419-025-08069-y)

Figure 2G

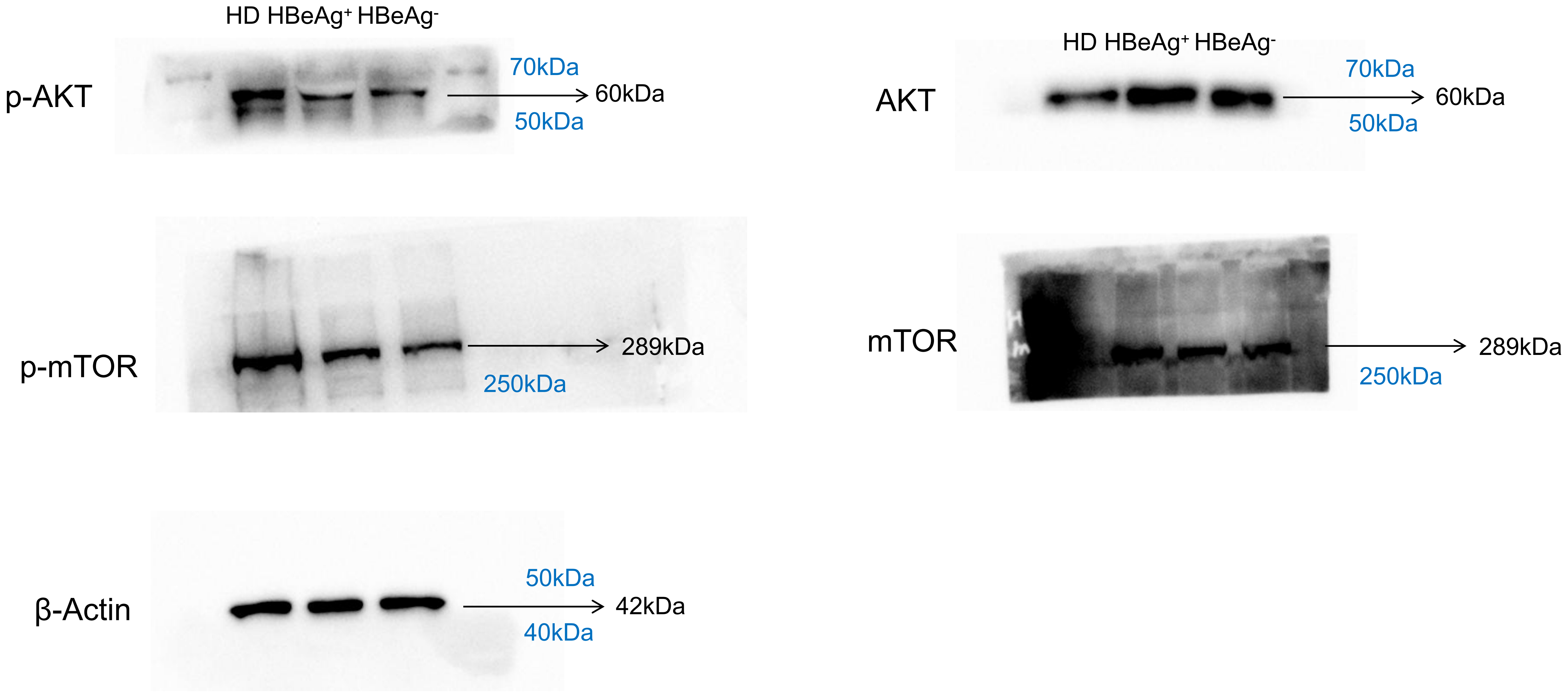

HD HBeAg+ HBeAg-
p-AKT
70kDa
60kDa
50kDa
HD HBeAg+ HBeAg-
AKT
70kDa
60kDa
50kDa
p-mTOR
289kDa
250kDa
mTOR
289kDa
250kDa
β-Actin
50kDa
42kDa
40kDa

Figure 2J

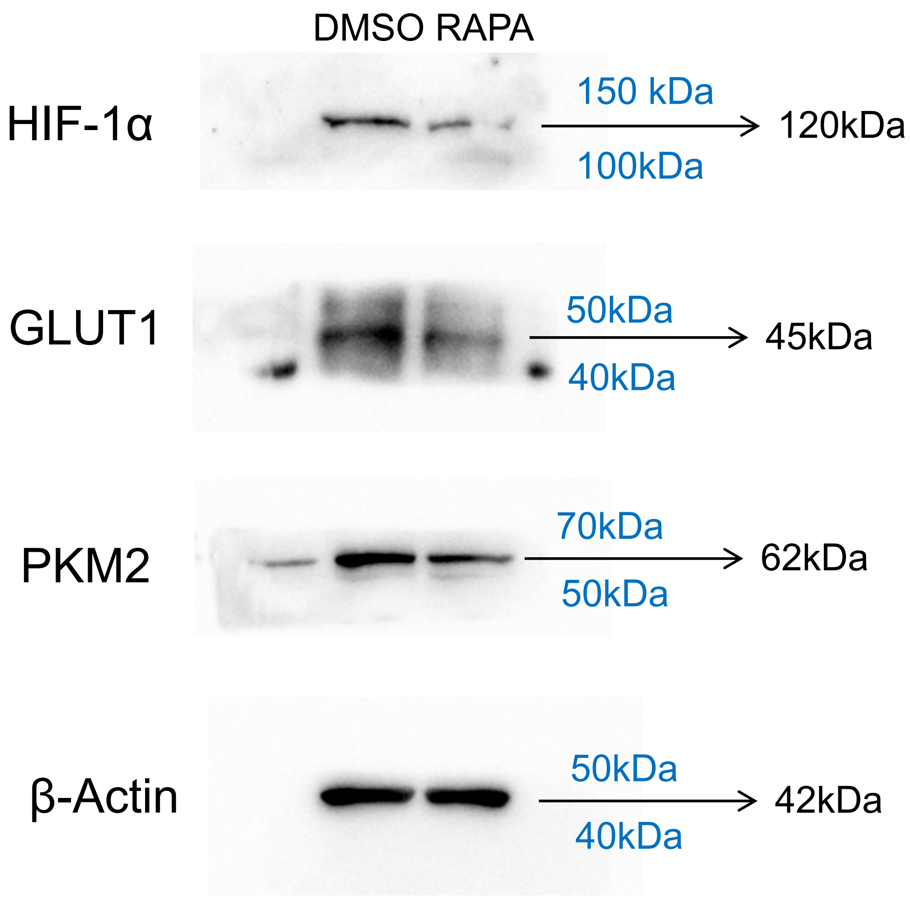
DMSO RAPA
HIF-1α
150 kDa
120kDa
100kDa
GLUT1
50kDa
45kDa
40kDa
PKM2
70kDa
62kDa
50kDa
β-Actin
50kDa
42kDa
40kDa

Figure 3D

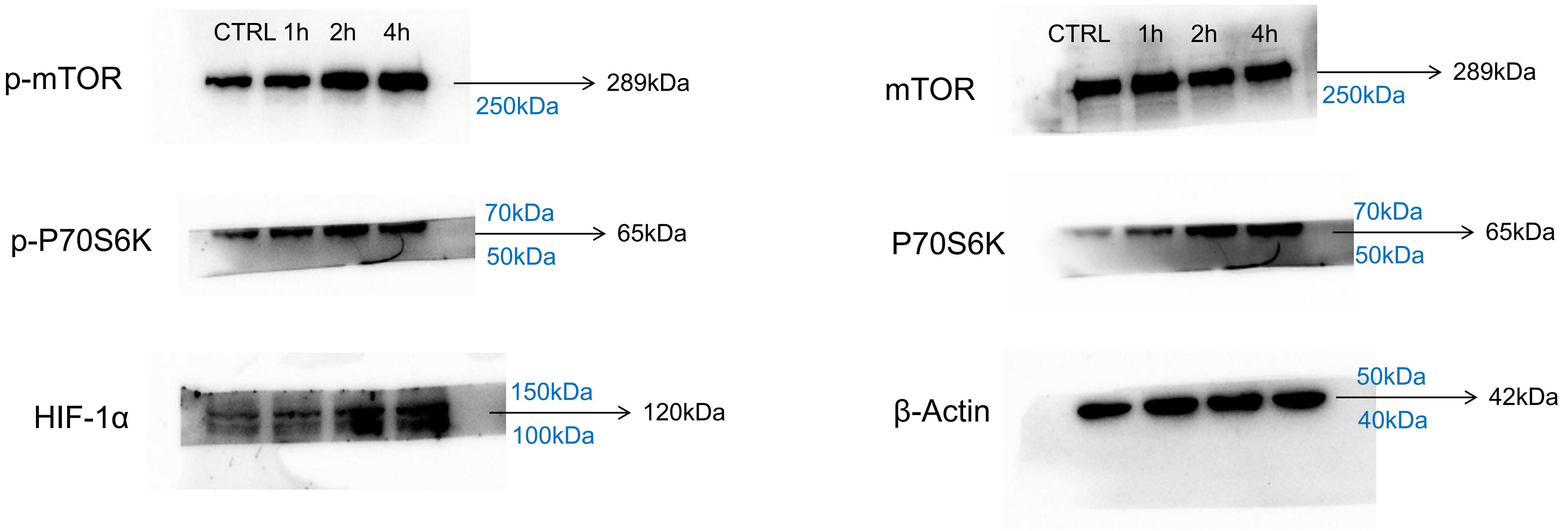

CTRL 1h 2h 4h
p-mTOR
289kDa
250kDa
p-P70S6K
70kDa
65kDa
50kDa
HIF-1α
150kDa
120kDa
100kDa
CTRL 1h 2h 4h
mTOR
289kDa
250kDa
P70S6K
70kDa
65kDa
50kDa
β-Actin
50kDa
42kDa
40kDa

Figure 3E

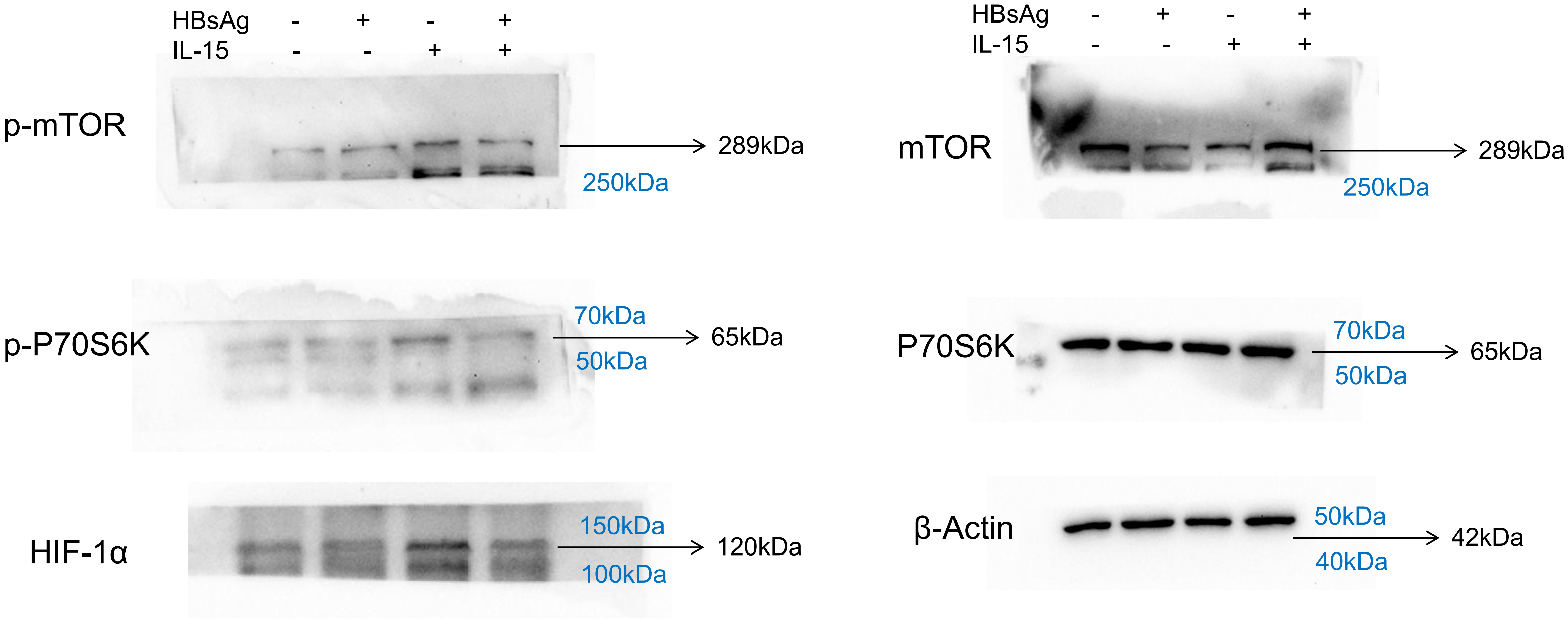
HBsAg - + - +
IL-15 - - + +
p-mTOR
289kDa
250kDa
p-P70S6K
70kDa
65kDa
50kDa
HIF-1α
150kDa
120kDa
100kDa
HBsAg - + - +
IL-15 - - + +
mTOR
289kDa
250kDa
P70S6K
70kDa
65kDa
50kDa
β-Actin
50kDa
42kDa
40kDa

Figure 5A

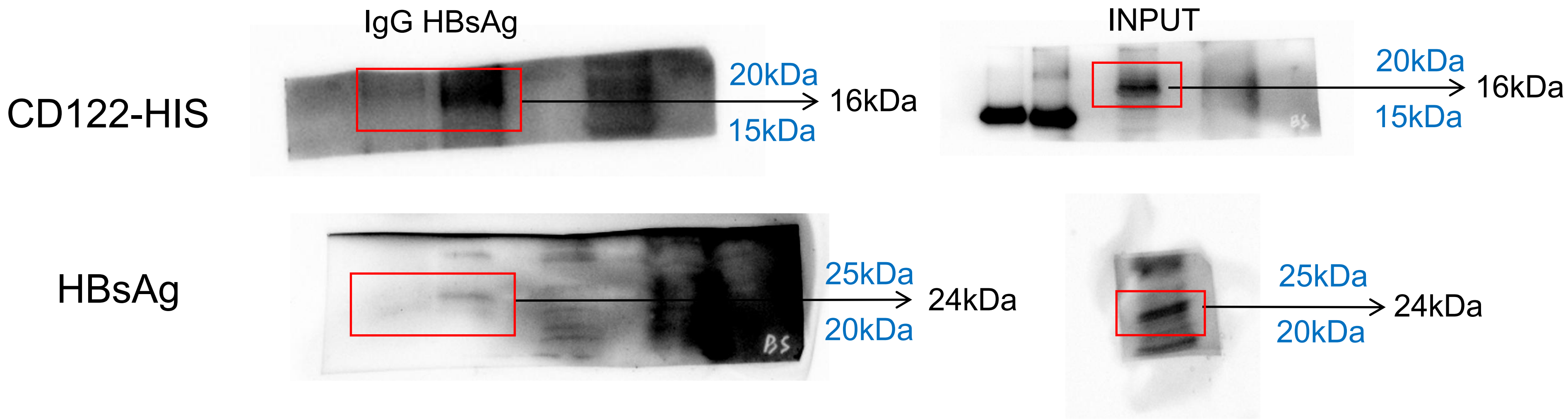
IgG HBsAg
INPUT
CD122-HIS
20kDa
16kDa
15kDa
20kDa
16kDa
15kDa
HBsAg
25kDa
24kDa
20kDa
25kDa
24kDa
20kDa

Figure 5B

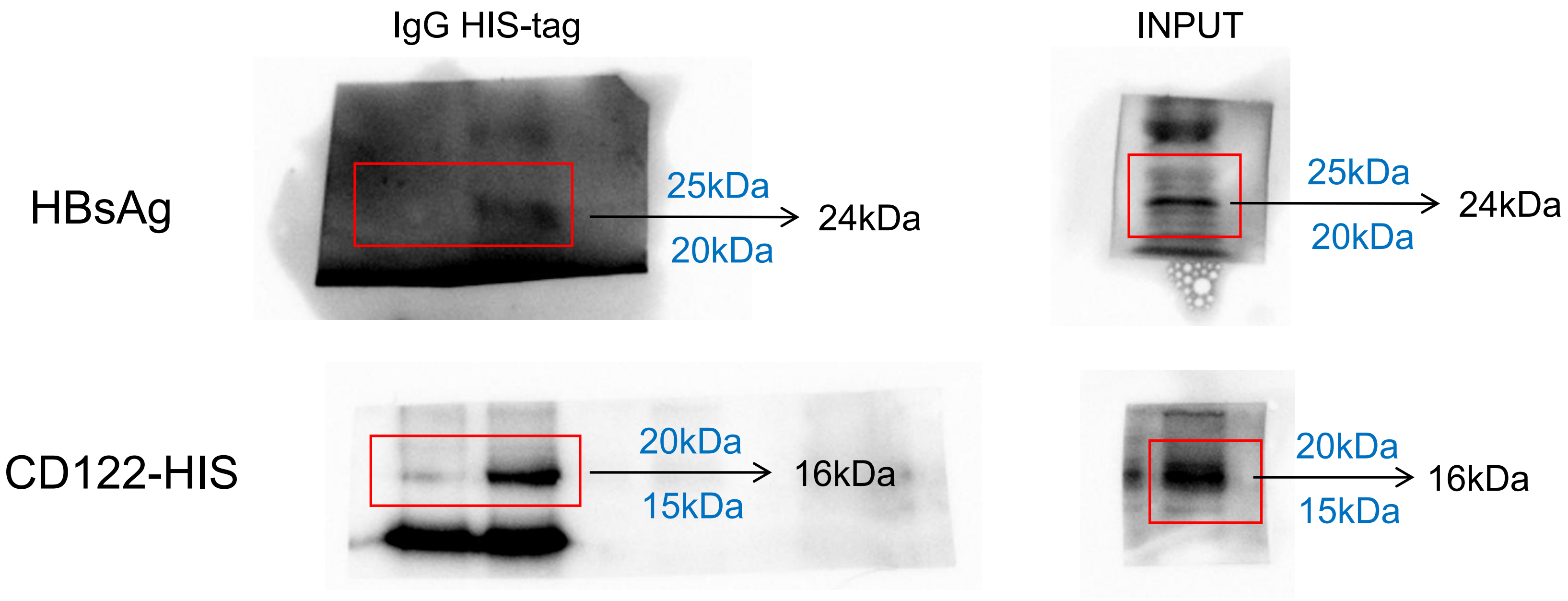
IgG HIS-tag
INPUT
HBsAg
25kDa
24kDa
20kDa
25kDa
24kDa
20kDa
CD122-HIS
20kDa
16kDa
15kDa
20kDa
16kDa
15kDa

Figure 5C

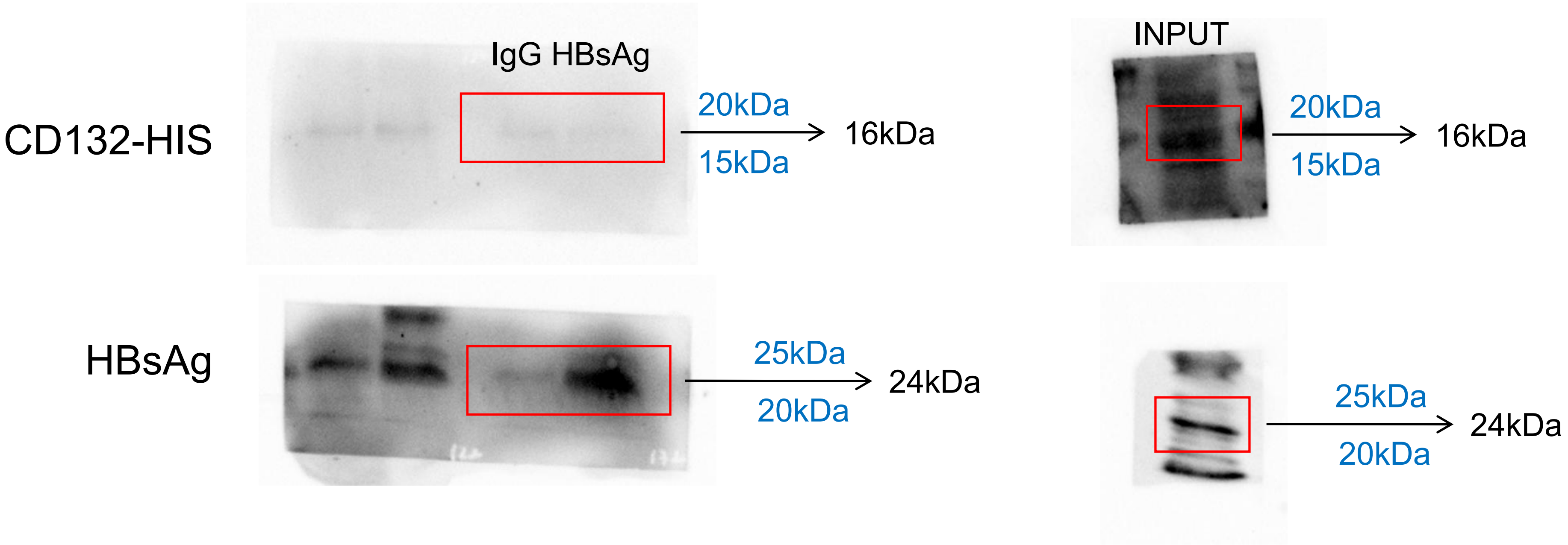
IgG HBsAg
CD132-HIS
20kDa
16kDa
15kDa
HBsAg
25kDa
24kDa
20kDa
INPUT
20kDa
16kDa
15kDa
25kDa
24kDa
20kDa

Supplementary Figure 2A

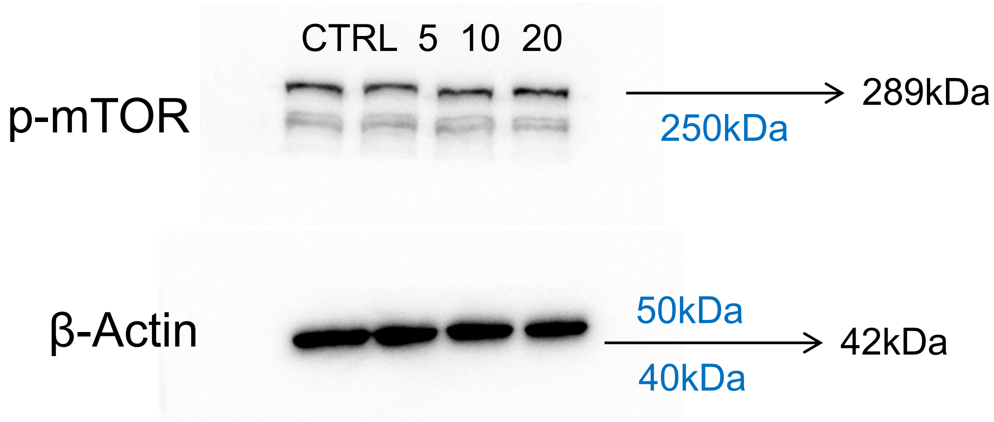

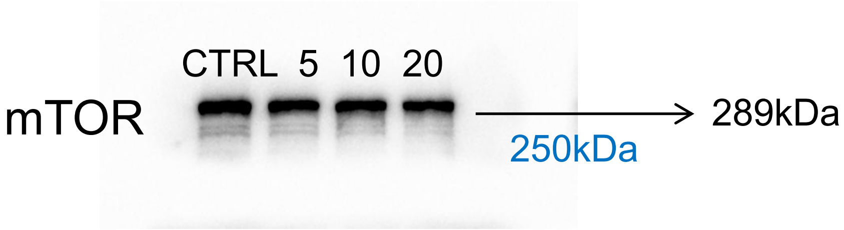

Supplementary Figure 4A

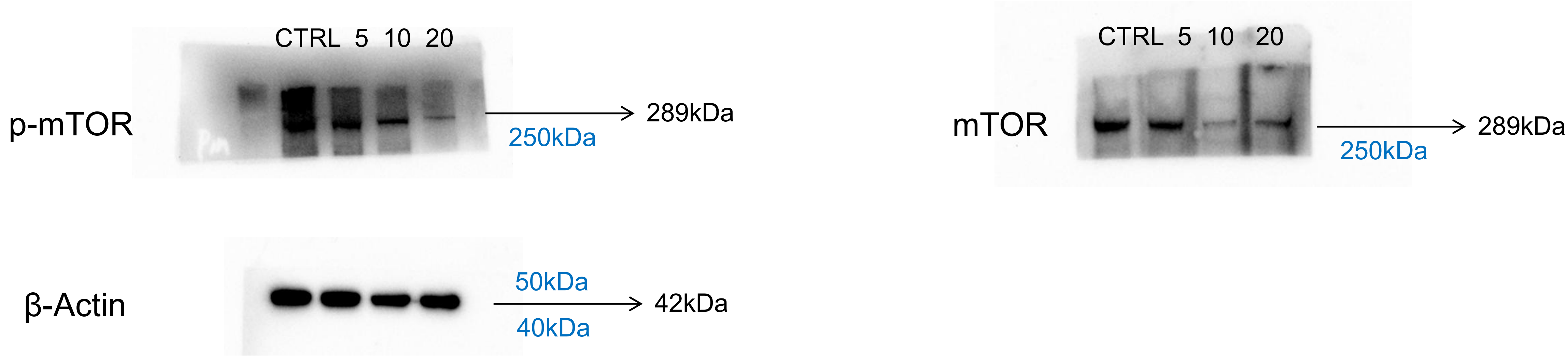

Supplement: Supplementary file 2 — Full and uncropped western blots [file 41419_2025_8069_MOESM2_ESM.pdf]
